# MicroRNA-143 targets ERK5 in granulopoiesis and predicts outcome of patients with acute myeloid leukemia

**DOI:** 10.1038/s41419-018-0837-x

**Published:** 2018-07-26

**Authors:** Jens-Uwe Hartmann, Daniela Bräuer-Hartmann, Miroslava Kardosova, Alexander A. Wurm, Franziska Wilke, Cindy Schödel, Dennis Gerloff, Christiane Katzerke, Rosanna Krakowsky, Carolina Yaeko Namasu, Marius Bill, Sebastian Schwind, Carsten Müller-Tidow, Dietger Niederwieser, Meritxell Alberich-Jorda, Gerhard Behre

**Affiliations:** 1 Division of Hematology and Oncology, Leipzig University Hospital, Leipzig, Germany; 2 Institute of Molecular Genetics of the ASCR, v.v.i. Laboratory of Haematooncology, Prague, Czech Republic; 3 Department of Internal Medicine V, University of Heidelberg, Heidelberg, Germany

## Abstract

Hematopoiesis, the formation of blood cells from hematopoietic stem cells (HSC), is a highly regulated process. Since the discovery of microRNAs (miRNAs), several studies have shown their significant role in the regulation of the hematopoietic system. Impaired expression of miRNAs leads to disrupted cellular pathways and in particular causes loss of hematopoietic ability. Here, we report a previously unrecognized function of miR-143 in granulopoiesis. Hematopoietic cells undergoing granulocytic differentiation exhibited increased miR-143 expression. Overexpression or ablation of miR-143 expression resulted in accelerated granulocytic differentiation or block of differentiation, respectively. The absence of miR-143 in mice resulted in a reduced number of mature granulocytes in blood and bone marrow. Additionally, we observed an association of high miR-143 expression levels with a higher probability of survival in two different cohorts of patients with acute myeloid leukemia (AML). Overexpression of miR-143 in AML cells impaired cell growth, partially induced differentiation, and caused apoptosis. Argonaute2-RNA-Immunoprecipitation assay revealed ERK5, a member of the MAPK-family, as a target of miR-143 in myeloid cells. Further, we observed an inverse correlation of miR-143 and ERK5 in primary AML patient samples, and in CD34^+^ HSPCs undergoing granulocytic differentiation and we confirmed functional relevance of ERK5 in myeloid cells. In conclusion, our data describe miR-143 as a relevant factor in granulocyte differentiation, whose expression may be useful as a prognostic and therapeutic factor in AML therapy.

## Introduction

MicroRNAs (miRNAs) are a class of small non-coding RNAs (ncRNAs), ∼ 19–25 nucleotides in length, which can inhibit the translation or induce the destabilization and/or degradation of their mRNA targets, usually by binding in an incomplete manner to the 3′ untranslated region (3′ UTR) of their respective targets^1^. Since their initial discovery, miRNAs have been found to play important roles in proliferation, differentiation, and apoptosis^2–4^. miRNAs have also been implicated in all stages of hematopoiesis including maintenance of hematopoietic stem cells (HSCs) and differentiation into mature effector cells^5,6^. We and others have shown that miRNAs play a key role as oncogenes^7–9^ or tumor suppressors^10–12^ in leukemia, the malignant transformation of hematopoiesis.

Acute myeloid leukemia (AML) as a very aggressive leukemic subtype is characterized by a large genetic heterogeneity and the presence of immature abnormal myeloid progenitor cells in the bone marrow^13^. Despite improvements in diagnosis and therapy, the 5-year survival rate of adult AML patients is only 30% (http://seer.cancer.gov). Diagnostic strategies continuously aim to identify novel prognostic markers such as gene mutations and DNA methylation to improve therapy options for patients^14^. In this context, abnormal expression of different miRNAs has been detected in distinct AML subtypes leading to activation or inhibition of essential pathways in leukemogenesis^15^. However, the function of individual miRNAs during normal and malignant hematopoiesis and their role as prognostic markers remains largely unknown.

miR-143 is an miRNA commonly seen to be downregulated in a variety of cancers, including hematopoietic malignancies^16,17^. Several studies implicate an important role of miRNA-143 to promote differentiation and to inhibit proliferation since it targets a number of cellular factors and pathways involved in transcription^18–20^. miR-143 is shown to target several tumor-associated factors and thereby interfere with fundamental cellular processes often found deregulated in cancer^21–23^. Due to this, miR-143 could have been described as tumor suppressor and prognostic marker in a wide range of tumors^24–26^.

ERK5 (extracellular signal-regulated kinase 5; MAPK7; mitogen-activated protein kinase 7) as a part of the MEK/ERK-pathway^27^ is a verified miR-143 target in solid cancers^28–30^. The transcription factor ERK5 is a central mediator of cell survival, proliferation, differentiation, and apoptotic regulation of normal cells^31–33^. Deregulation and activation of ERK5 has been shown to be a frequent event in the onset and progression of cancer^34–36^. Furthermore, recent publications describe the involvement of ERK5 in therapy response, including leukemia^37,38^. The interaction between the tumor suppressor miR-143 and oncogenic ERK5 signaling is well characterized in solid cancers, but their interplay is rather unknown in the background of AML.

In the present study, we explore the role of miR-143 in hematopoietic differentiation and AML. We found miR-143 to be upregulated during granulocytic differentiation of primary human CD34^+^ stem/progenitor cells (HSPCs), primary acute promyelocytic leukemia (APL) patient samples, and various AML cell lines. Furthermore, we demonstrate the importance of miR-143 expression for granulocytic differentiation in vitro and in vivo. In line with this, we identified high miR-143 expression as a favorable prognostic factor in AML. By ectopic expression of miR-143, we showed ERK5, an important member of the MAP-kinase pathway, as a target of miR-143. This finding is supported by inverse correlation of miR-143 expression and ERK5 protein in CD34^+^ HSPCs and AML patient samples. Taken together, our data depict an important role of miR-143 in normal granulocytic differentiation and treatment response in AML and thereby provides a novel source for clinical applications of miRNAs in the context of myeloid leukemia.

## Results

### miR-143 is upregulated during granulopoiesis in vitro and in vivo

**Fig. 1:**
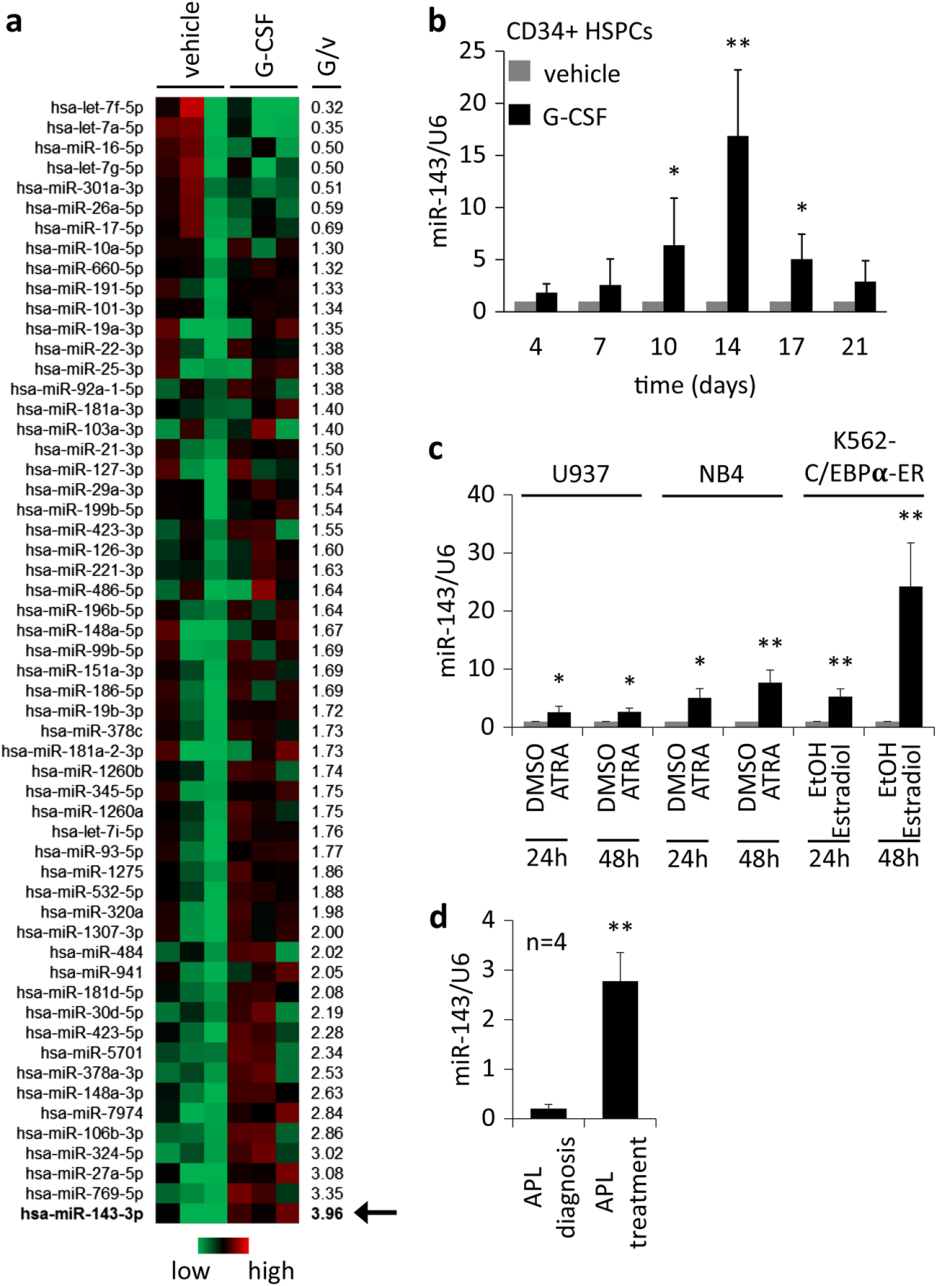
miR-143 is upregulated during granulopoiesis in vitro and in vivo. **a** Heat map of differentially regulated microRNAs in G-CSF or vehicle-treated human CD34^+^ hematopoietic stem cells (HSPCs). miRNA expression pattern was evaluated by next-generation sequencing (NGS), 7 days after treatment. The miRNA expression was calculated to the median expression of each miRNA. Numbers beside the heat map represent the ratio G-CSF/vehicle from all three experiments. miR-143 expression is marked with an arrow (red = high, green = low). **b** qPCR analysis of miRNA-143 expression in G-CSF or vehicle (H_2_O) treated CD34^+^ HSPCs over 21 days. **c** ATRA-treated NB4 and U937 cells as well as β-estradiol treated K562-C/EBPα-ER at indicated time points. Bars represent the mean ± SD from at least three independent experiments (**b**, **c**). **d** qPCR analysis of miR-143 expression in primary APL patient bone marrow or peripheral blood samples at diagnosis and during ATRA treatment. Bars represent the mean ±SD [*n* = 4]. *P ≤ 0.05; **P ≤ 0.01, unpaired two-tailed *t*-tests

Even though several miRNAs have been shown to be regulated in granulopoiesis, whether miRNAs are downstream targets of cytokines and how this regulation is instrumental in granulopoiesis is not known. In order to identify differentially expressed miRNAs during granulocytic differentiation, we treated primary human CD34^+^ HSPCs with G-CSF or vehicle (H_2_O). Next-generation sequencing (NGS) was performed at day 7 of treatment and revealed several differentially regulated miRNAs, including miR-143 as the most upregulated (3.96-fold); (Fig. 1a). Differentiation status and viability was assessed by FACS-based measurement of CD15 and morphology (Supplementary Fig. 1a–c). To confirm NGS data, miR-143 expression was measured in G-CSF treated CD34^+^ HSPCs by qPCR. We observed a continuous increase of miR-143 levels until day 14 followed by a decline thereafter (Fig. 1b). To exclude cell-specific effects, we analyzed miR-143 expression in different hematopoietic cell lines (U937, NB4, and K562-C/EBPα-ER) undergoing granulocytic differentiation. All cells showed a significant upregulation of miR-143 expression at the indicated time points compared to the control (Fig. 1c). Granulocytic differentiation was confirmed by morphology and FACS-based measurement of CD11b (Supplementary Fig. 1d-f). Since ATRA causes granulocytic differentiation, we also analyzed bone marrow and blood samples from APL-patients undergoing ATRA-based therapy. qPCR analysis revealed the ATRA-dependent upregulation of miR-143 after treatment (Fig. 1d, Supplementary Table 1).

### miR-143 is important for granulocytic differentiation

**Fig. 2:**
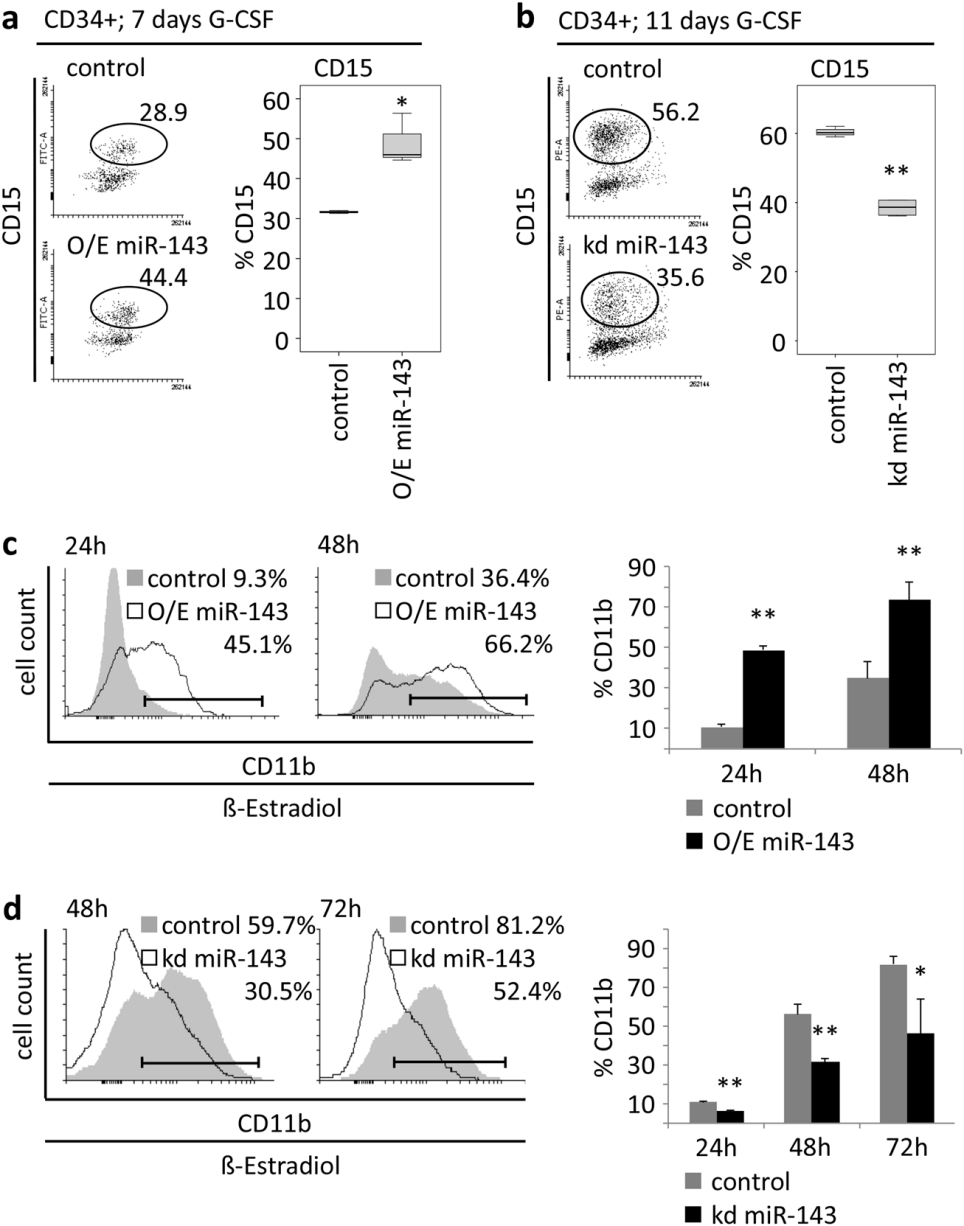
miR-143 is necessary for granulocytic differentiation. **a, b** FACS analysis of CD15 expression of G-CSF-treated CD34^+^ HSPCs (**a**) 7 days after stable overexpression (**b**) 11 days after stable knockdown of miR-143. **c, d** FACS analysis of CD11b expression at indicated time points in K562-C/EBPα-ER cells (**c**) after transient miR-143 overexpression or (**d**) stable miR-143 knockdown and treatment with β-estradiol. Numbers in dot blots indicate percentage of CD15^+^ or CD11b^+^ cells. Bars in diagrams represent the mean ± SD from three independent experiments. *P* values are relative to control. *P ≤ 0.05; **P ≤ 0.01, unpaired two-tailed *t*-tests

To address the role of miR-143 in hematopoietic differentiation, we performed stable overexpression (O/E) or knockdown (kd) of miR-143 in HSPCs and hematopoietic cell lines. Overexpression of miR-143 in CD34^+^ HSPCs followed by G-CSF treatment resulted in accelerated expression of CD15 at day 7 (Fig. 2a), whereas the knockdown of miR-143 caused a decrease in CD15 expression at day 11 (Fig. 2b). Transient overexpression of miR-143 in the myeloid cell line K562-C/EBPα-ER followed by induction of granulocytic differentiation with β-estradiol also caused an accelerated differentiation compared to control cells (Fig. 2c, Supplementary Fig. 2a). In contrast to this, lentiviral reduction of miR-143 levels in K562-C/EBPα-ER cells resulted in a reduced CD11b expression upon β-estradiol treatment compared to the control (Fig. 2d, Supplementary Fig. 2b). To confirm these data, we performed knockdown of miR-143 in NB4 and U937 cells and observed comparable results (Supplementary Fig. 2c, d).

### Stable knockdown of miR-143 leads to a reduction of granulocytic cells in mice

**Fig. 3:**
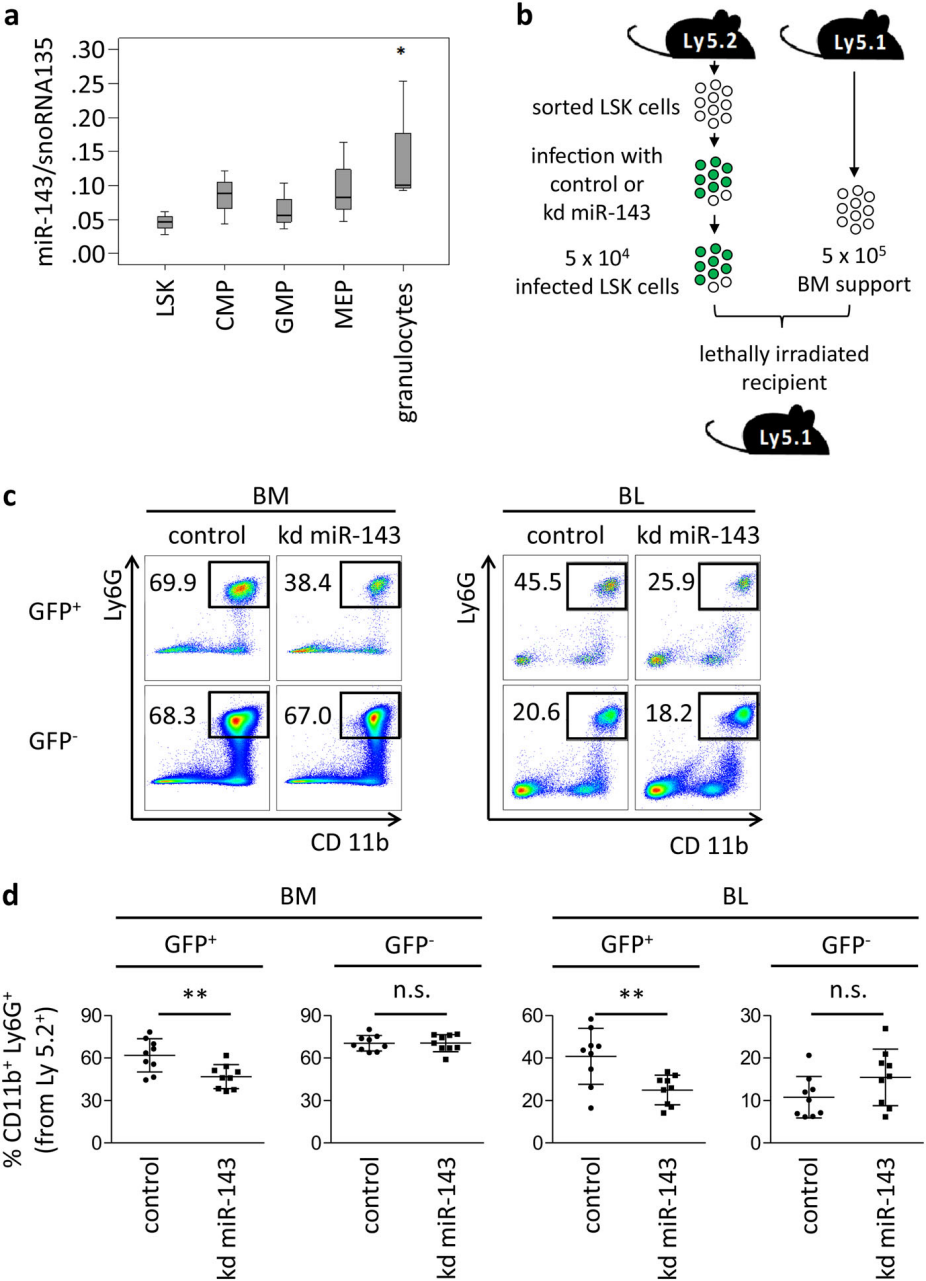
Stable knockdown of miR-143 leads to reduced granulocytic differentiation in vivo. **a** qPCR for miR-143 expression in sorted mouse bone marrow cell subpopulations. Bars represent the mean ± SD from at least three independent cell sortings. Bars are represented as ΔCt values. *P* value of granulocytes is relative to all other analyzed samples. *P < 0.05. **b** Schematic overview of the LSK-based knockdown (kd) transplantation model. LSK cells sorted from C57Bl/6 Ly5.2^+^ mice were transduced with miRZip-143 (kd miR-143) or miRZip-control (control) lentivector-based constructs. Of note, miRZip construct contains a GFP reporter. Next, non-sorted LSK cells (containing a mixture of infected and non-infected cells) were mixed with 5 × 10^5^ total bone marrow cells isolated from C57Bl/6 Ly5.1^+^ congenic mice and transplanted into lethally irradiated C57Bl/6 Ly5.1^+^ recipients by tail vein injection. Importantly, the infection efficiency was similar in miRZip-143 and miRZip-control. Analysis of recipient mice was performed 3 weeks after transplantation. **c** Flow cytometric analysis representative plots from BM and BL of recipient mice transplanted with control (left panels) or miR143 knockdown (kd miR-143, right panels) LSK cells. Plots were generated by gating on donor-derived Ly5.2^+^ cells, and subsequently divided into GFP^+^ (infected) and GFP^−^ (non-infected) cells. Black boxes indicate percentage of mature Ly6G^+^/CD11b^+^ granulocytes. The percentage of donor-derived mature granulocytes in BM and BL of recipient mice were reduced when miR-143 levels were downregulated (GFP^+^ kd miR-143) in comparison to control (GFP^+^ control). GFP^−^fraction (lower panels) served as an additional negative control. Dead cells were excluded from analysis by Hoechst 33258 staining. **d** Quantification of the flow cytometry analysis described in panel **c**. *Y*-axis indicates the percentage of mature Ly6G^+^/CD11b^+^ granulocytes derived from donor Ly5.2^+^ cells. Bars represent the mean ± SD from two independent experiments, *n* = 9 (control) or 10 (kd miR-143) mice, **P < 0.01, unpaired two-tailed *t*-tests were used to assess statistical significance. LSK, Lin^−^ Sca-1^+^ cKit^+^ cells; CMP, common myeloid progenitors; GMP, granulocyte–macrophage progenitors; MEP, megakaryocyte erythroid progenitors and granulocytes*;* Kd, knockdown; BM, bone marrow; BL, blood

To investigate miR-143 expression during normal granulopoiesis, we sorted distinct murine bone marrow populations from C57BL/6NCrl mice. qPCR analysis showed a steady increase of miR-143 expression according to the differentiation progress, with the highest miR-143 expression levels in mature granulocytes (Fig. 3a). Next, we investigated whether downregulation of miR-143 would impair granulocytic differentiation in vivo. Therefore, we performed murine bone marrow transplantation experiments with manipulated lineage^−^ Sca-1^+^ cKit^+^ (LSK) cells, population enriched for hematopoietic stem and progenitor cells (Fig. 3b). Sorted LSK cellsisolated from C57BL/6NCrl mice (Ly5.2) were transduced with pseudoviral particles carrying lentivector for miR-143 knockdown (miRZip-143) or control vector (miRZip-control). Importantly, miRZip vector contains a GFP reporter that allows tracking of infected cells. Since we did not sort GFP^+^ cells prior to transplantation, the donor-derived Ly5.2 population consists of a mixture of transduced (GFP^+^) and untransduced (GFP^−^) cells. Efficiency of transduction was similar in both groups as measured by assessing GFP positivity 48 h post transduction (data not shown). Recipient mice were killed and blood and bone marrow were analyzed 3 weeks after transplantation. As expected, we did not observe changes in the engraftment of total Ly5.2^+^ cells or contribution of GFP^+^ donor cells to the hematopoietic system of the recipient mice (Supplementary Fig. 3a, b). However, flow cytometry analysis showed a significant reduction of CD11b^+^/Ly6G^+^ mature granulocytes in the GFP^+^ fraction of the cells with miR-143 knockdown (kd miR-143) compared to GFP^+^ control cells both in peripheral blood and bone marrow of recipient mice (Fig. 3c). No effect was observed in the GFP^−^ non-infected fraction. Of note, the percentage of mature CD11b^+^/Ly6C^+^ monocytes were not affected (Supplementary Fig. 3c). To determine cell morphology of the donor-derived cells, we sorted Ly5.2^+^/GFP^+^ cells from BM of recipient mice, cytospun them, and performed May–Grünwald/Giemsa staining. In line with the flow cytometric data, we observed reduced numbers of mature neutrophils in mice transplanted with miR-143 knockdown cells compared to the controls (Supplementary Fig. 3d, e). Altogether, we demonstrated that miR-143 is gradually expressed during granulocytic differentiation in all developmental stages, detected the highest levels of miR-143 in mature granulocytes, and showed that miR-143 knockdown in LSK cells partially blocks granulocytic differentiation in vivo.

### High miR-143 expression is a prognostic factor for favorable outcome in AML

**Fig. 4:**
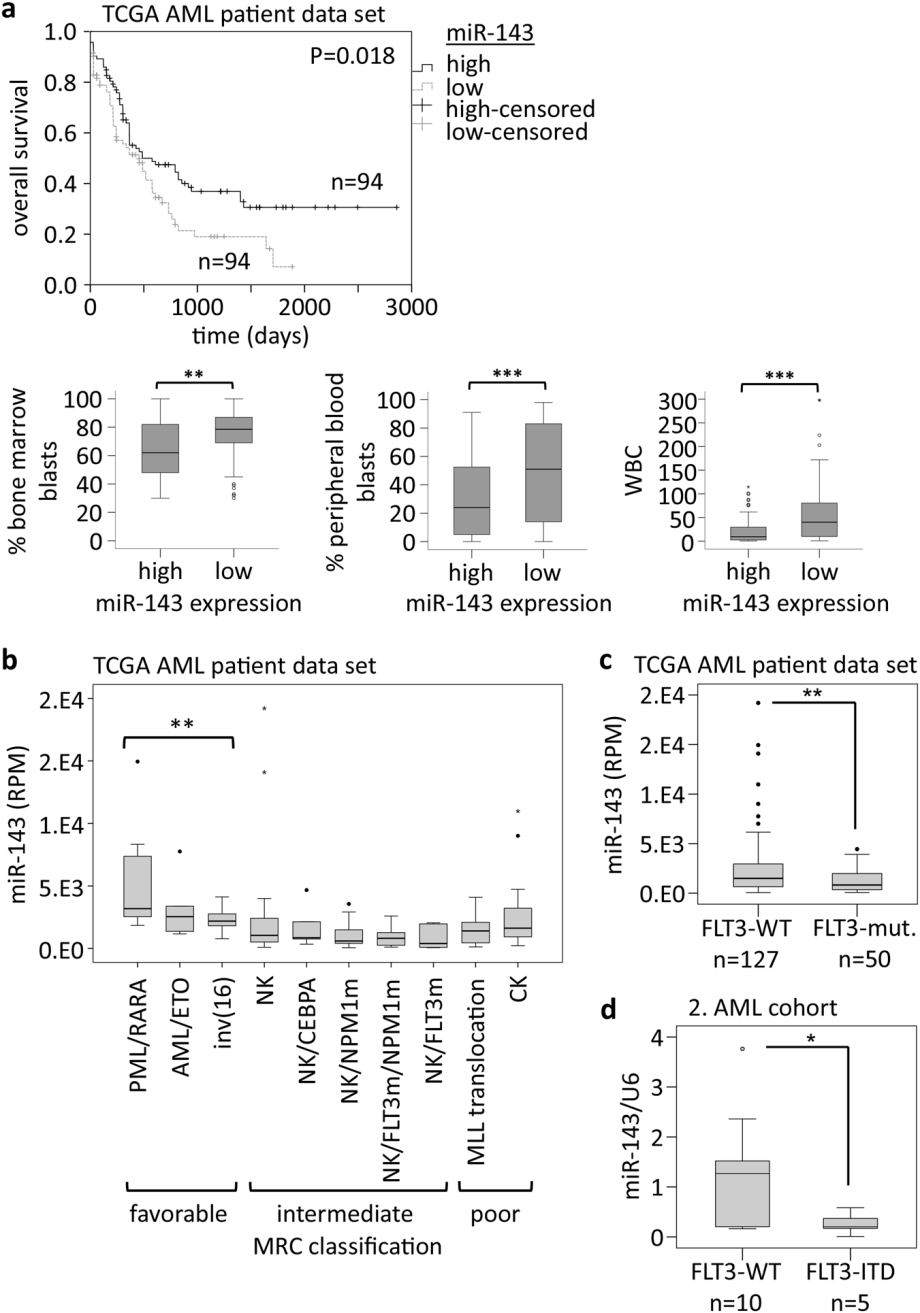
miR-143 is a good prognostic factor in AML. **a** Overall survival of AML patients (TCGA Research Network), according to the dichotomized miR-143 expression; (*n* = 188; Mann–Whitney-*U* test); (upper panel). Bone marrow blast count (BM blasts; left), peripheral blood blast count (PB blasts; middle), and number of white blood cells (WBC, right) in AML patients (TCGA Research Network), according to the dichotomized miR-143 expression (lower panel). Bars represent the median ± SD [high miR-143, *n* = 94; low miR-143, *n* = 94]. **P < 0.01; ***P < 0.001, unpaired two-tailed *t*-tests. **b** miR-143 expression in different AML subgroups classified according to the MRC (the British Medical Research Council); (TCGA). **c** miR-143 expression in AML patients with and without FLT3-mutations; (TCGA). Bars represent the median ± SD [FLT3-WT, *n* = 127; FLT3-mut. *n* = 50], *P* values are relative to FLT3-WT. **P < 0.01, unpaired two-tailed *t*-tests. **d** miR-143 expression in AML patients with wildtype FLT3 or a FLT3-ITD mutation (2. AML cohort). Bars represent the median ± SD [FLT3-WT, *n* = 10; FLT3-ITD, *n* = 5], *P* values are relative to FLT3-WT. *P < 0.05, unpaired two-tailed *t*-tests

To investigate the impact of miR-143 on AML prognosis, we analyzed miR-143 expression in the TCGA AML patient data set (GDC Data Portal). AML patients with high miR-143 expression showed a significantly longer overall survival (OS) than AML patients with low miR-143 expression. According to this, AML patients with high miR-143 expression had significantly lower percentages of blasts in peripheral blood and bone marrow, as well as significantly lower WBC counts than AML patients with low miR-143 expression (Fig. 4a, Supplementary Table 2). To verify our data, we analyzed miR-143 expression in a second cohort, capturing 47 AML patients, who received non-myeloablative (NMA) conditioning before allogeneic HSC transplantation. In this cohort, high miR-143 expression was associated with a significantly lower WBC count and lower percentages of blasts in peripheral blood, but not in bone marrow at diagnosis (Supplementary Fig. 4a). Whereas OS was not different in this patient cohort, AML patients with high miR-143 expression showed a significantly longer event-free survival than patients with low miR-143 expression (Supplementary Fig. 4b). Further, we investigated miR-143 expression in different cytogenetic AML subclasses of the TCGA data set and observed a significantly higher miR-143 expression in the MRC (British Medical Research Council) classified favorable risk group compared to the intermediate-risk and poor-risk group (Fig. 4b). Here, AML patients with an FLT3 receptor mutation, a mutation connected to unfavorable prognosis^39^, showed the lowest miR-143 expression (Fig. 4b, c, Supplementary Table 2). qPCR analysis of a second AML patient set also showed that AML patients with an FLT3-ITD mutation showed significantly lower miR-143 expression levels compared to FLT3 wild type (Fig. 4d, Supplementary Table 3).

### miR-143 impairs growth, survival, as well as partial differentiation of leukemic cells

**Fig. 5:**
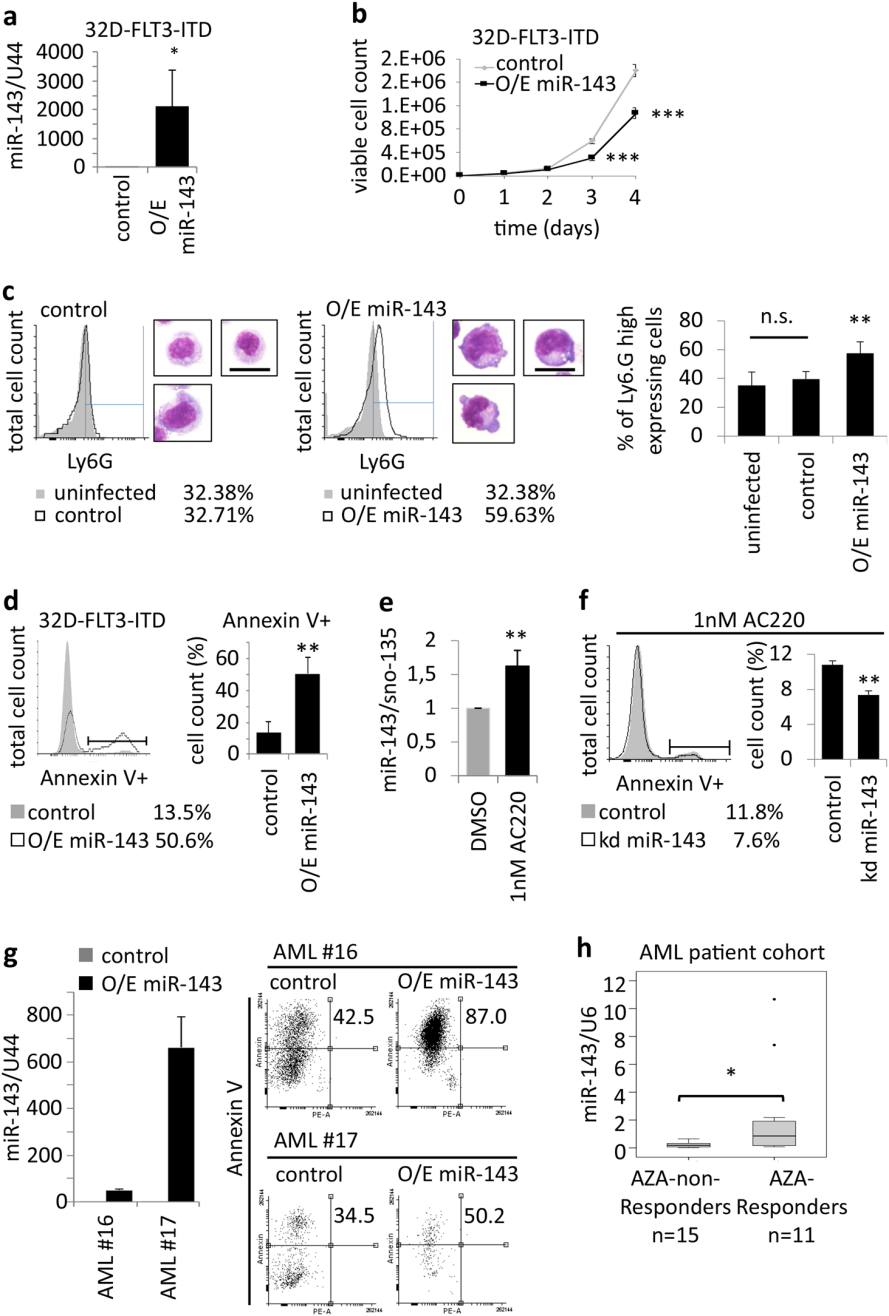
miR-143 impairs AML cell growth, survival, and in partial differentiation. **a** qPCR analysis of exogenous miR-143 expression in 32D-FLT3-ITD cells. Bars represent the mean±SD from three independent experiments. *P* values are relative to control U44. *P < 0.05; **P < 0.01, unpaired two-tailed *t*-tests. **b** Cell growth curve of 32D-FLT3-ITD cells stably overexpressing miR-143 or control. Values represent mean ± SD [*n* = 4]. ***P < 0.001, unpaired two-tailed *t*-tests. **c** FACS analysis for Ly6G expression and representative morphology pictures of 32D-FLT3-ITD cells overexpressing miR-143 or control. Scale bar = 10 µm. The column chart (right) represents the mean of three independent experiments ± SD. *P* values are relative to control. **P ≤ 0.01, unpaired two-tailed *t*-tests. **d** FACS analysis for Annexin V-stained 32D-FLT-ITD cells overexpressing miR-143. Bars represent the mean ± SD from three independent experiments. *P* values are relative to control. **P < 0.01, unpaired two-tailed *t*-tests. **e** qPCR for miR-143 expression in 32D-FLT3-ITD cells treated with 1 nM AC220 for 24 h. Bars represent the mean ± SD from three independent experiments. *P* values are relative to control. **P < 0.01, unpaired two-tailed *t*-tests. **f** FACS analysis for Annexin V-stained 32D-FLT-ITD cells with stable knockdown of miR-143 or control after 24 h treatment with 1 nM AC220. Bars represent the mean ± SD from three independent experiments. *P* values are relative to control. **P < 0.01, unpaired two-tailed *t*-tests. **g** qPCR analysis of exogenous miR-143 expression in bone marrow cells of AML patients (left). Bars represented as technical replicates. FACS analysis of Annexin V-stained AML patient samples overexpressing miR-143 or control (right). Numbers in dot blots indicate the percentage of Annexin V-positive cells. **h** qPCR for miR-143 expression in AML patients undergoing 5-acazytidine therapy (AZA). Values represent the mean ± SD [non-responder, *n* = 15; responder, *n* = 11]. *P* values are relative to AZA-non-responders. *P < 0.05, unpaired two-tailed *t*-tests

To investigate miR-143 effects in leukemic cells, we overexpressed miR-143 in 32D cells stably expressing FLT3-ITD. miR-143 enrichment was confirmed by qPCR (Fig. 5a). Cell growth analysis showed that 32D-FLT3-ITD cells overexpressing miR-143 showed significantly less cell growth than the control cells (Fig. 5b). Furthermore, miR-143 overexpression resulted in a slight increase of the granulocytic surface marker Ly6G and a more mature morphology toward granulocytes (Fig. 5c, Supplementary Fig. 5). Additionally, we observed a significant increase in apoptotic cell number in miR-143 overexpressing 32D-FLT3-ITD compared to the control (Fig. 5d). Furthermore, the inhibition of FLT3 signaling by the potent FLT3 inhibitor AC220 (Quizartinib) leads to the induction of miR-143 expression in 32D-FLT3-ITD cells 24 h after treatment (Fig. 5e). In turn, stable inhibition of miR-143 in 32D-FLT3-ITD cells partially prevents AC220-mediated effects on apoptosis (Fig. 5f). To evaluate FLT3-ITD-independent effects of miR-143 on AML cells, we stably overexpressed miR-143 in freshly isolated AML patient bone marrow samples (Supplementary Table 4). FACS-based measurement of Annexin V-stained cells showed a significant increase in apoptosis in miR143 overexpressing cells compared to control cells (Fig. 5g). Finally, we also investigated miR-143 expression in bone marrow samples from AML patients receiving a 5-azacytidine-based therapy. qPCR analysis showed that patients who respond to the therapy had significantly higher miR-143 levels at diagnosis than patients who did not respond (Fig. 5h, Supplementary Table 5).

### ERK5 is a target of miR-143

**Fig. 6:**
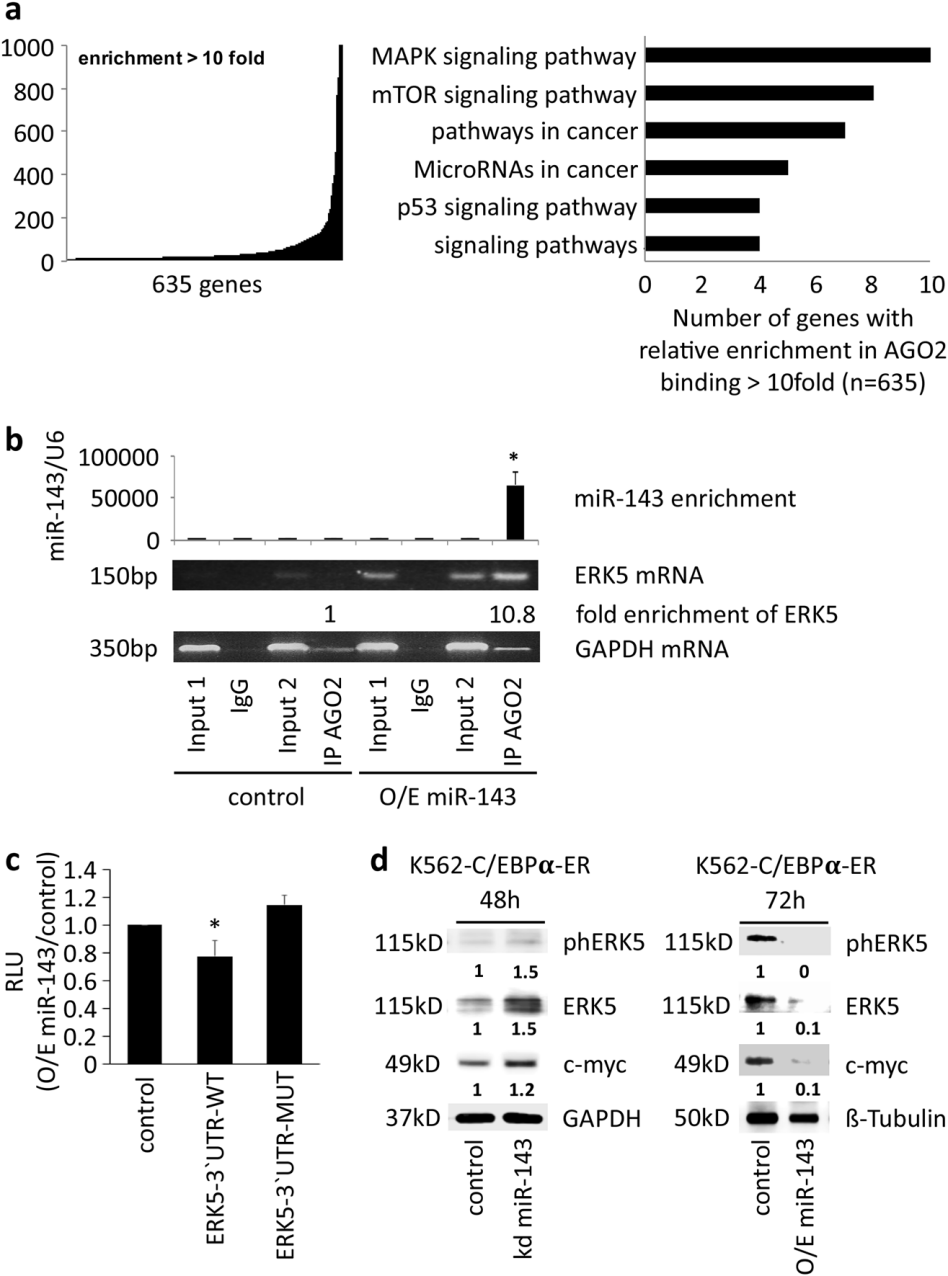
miR-143 induces ERK5 downregulation. **a** Enriched genes in miR-143 overexpressing K562-C/EBPα-ER cells after AGO-RIP. Genes detected by RIP-sequencing were plotted against their relative enrichment in AGO2 binding. A 10-fold enrichment in RIP samples was chosen as cut-off for identifying AGO2-bound mRNAs (left). KEGG pathway analysis of genes enriched after AGO-RIP (right). Data represent three independent experiments. **b** miR-143 and ERK5 mRNA enrichment in AGO-RIP samples. qPCR for miR-143 (upper panel). Bars of the diagram show the fold change of miR-143 expression compared to the input of the control infected cells. Values represent the mean ± SD from three independent experiments. *P* values are relative to control. *P < 0.05; unpaired two-tailed *t*-tests. Semiquantitative PCR for ERK5 mRNA and GAPDH as non-binding control in the AGO-RIP samples (lower panels). The gel pictures represent one example of three independent experiments. Numbers below the gel picture indicate the enrichment of ERK5 mRNA in the AGO-RIP of the miR-143 O/E sample compared to the AGO-RIP of the control sample. **c** Luciferase assay for direct miR-143 binding to the 3´UTR of ERK5. K562-C/EBPα-ER cells were cotransfected using 0.1 µg pRL, 1 µg of either pGL3-ERK5 3´ UTR-WT, pGL3-ERK5 3´UTR-MUT, or pGL3-C/EBPα 3´ UTR in combination with miR-143 or control mimics. Bars represent the luciferase activity for the corresponding vectors. Normalization was done by Renilla luciferase (pRL). Bars in the diagram represent the mean of 3 independent experiments ± SD. *P < 0.05; unpaired two-tailed *t*-tests. **d** Western blot for phosphorylated ERK5, ERK5, and c-Myc protein in K562-C/EBPα-ER cells after transient knockdown (left) and overexpression of miR-143 (right) at indicated time points. GAPDH and β-tubulin were used as control. The western blot pictures represent one example of three independent experiments. Numbers below the blots represent the ratio to the respective controls

Because miRNAs exert their function via targeting specific mRNAs, we used the argonaute 2-RNA-immunoprecipitation (AGO-RIP) technology to identify miR-143 target mRNAs in myeloid cells. miR-143 was stably overexpressed in K562-C/EBPα-ER cells and AGO-RIP followed by NGS for mRNAs was performed (Supplementary Fig. 6a). We identified a total number of 635 mRNAs, which showed at least 10-fold increased binding to the argonaute 2 protein in the miR-143 overexpressing cells compared to the control cells (Fig. 6a, left panel). Using KEGG pathway analysis, we identified several pathways targeted by miR-143, including the MAP-kinase-signaling pathway as the most targeted pathway (Fig. 6a, right panel). The transcription factor extracellular signal-regulated kinase 5 (ERK5), also known as mitogen-activated protein kinase 7 (MAPK7), is a main component of the MAPK-pathway^27^ and was significantly enriched in the AGO-RIP NGS screen (data not shown). The ERK5 3´ UTR carries one conserved miR-143 binding site (Supplementary Fig. 6b). By semiquantitative PCR, we confirmed the NGS data and observed the enrichment of ERK5 mRNA in the AGO-RIP samples from miR-143 overexpressing cells compared to control cells and IgG antibody control (Fig. 6b). To prove the direct binding of miR-143 to the predicted binding site in the 3´ UTR of ERK5 (Supplementary Fig. 6b), we performed a luciferase assay. Therefore, we co-transfected pGL3-constructs containing either the wild type ERK5-3´ UTR, the ERK5-3´ UTR with mutated miR-143 binding site or control (a shorten part of the C/EBPα-3´ UTR without any predicted miR-143 binding site)^9^ in combination with miR-143 mimics or control oligos as well as a pRL construct. Luciferase activity measurement revealed the direct binding of miR-143 to the predicted binding site (Fig. 6c). In addition, Western blot analysis showed miR-143-dependent changes in the protein levels of ERK5, activated ERK5, and the ERK5-downstream target c-Myc in K562-C/EBPα-ER and NB4 cells (Fig. 6d, Supplementary Fig. 6c, d).

### ERK5 protein inversely correlates with miR-143 expression, interferes with granulocytic differentiation, and is relevant for myeloid cell survival

**Fig. 7:**
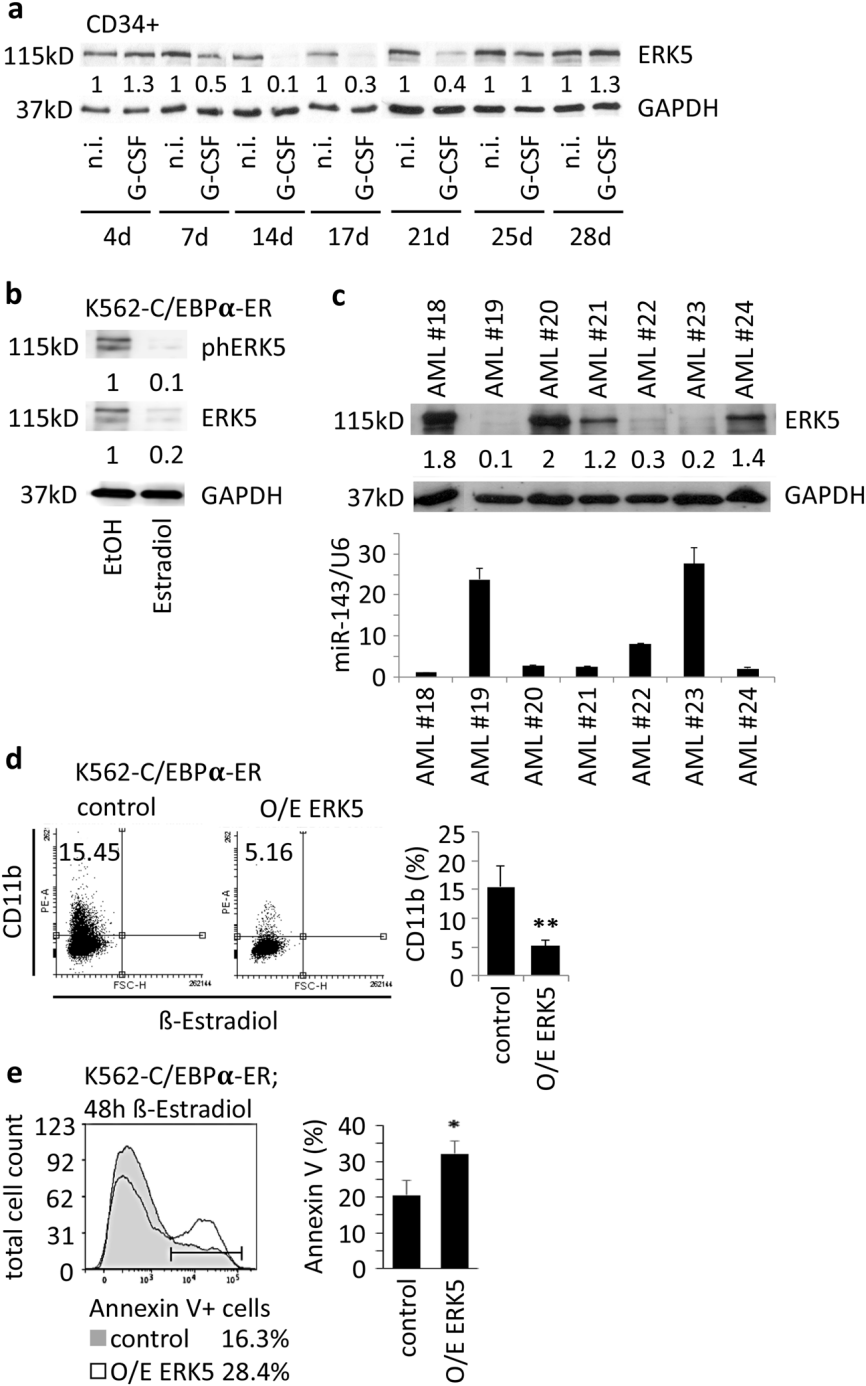
ERK5 protein inversely correlates with miR-143 expression, interferes with granulocytic differentiation, and is relevant for myeloid cell survival. **a** Western blot for ERK5 protein in G-CSF-treated CD34^+^ HSPCs at indicated time points. The western blot pictures represent one example of three independent experiments. Numbers below the blots represent the mean ratio to the respective controls. **b** Western blot for phosphorylated ERK5 and ERK5 protein in K562-C/EBPα-ER cells 48 h after β-estradiol treatment. The western blot pictures represent one example of three independent experiments. Numbers below the blots represent the ratio to the respective controls. **c** Western blot for ERK5 protein (upper panel) and qPCR for miR-143 expression (lower panel) in primary AML patient samples. GAPDH was used for normalization. The western blot pictures represent one example of three technical replicates. For AML patient samples the ratios were divided by the mean of all samples. The bars in the diagram (lower panel) summarize the mean ± SD of three technical replicates. **d** Transient overexpression of ERK5 in K562-C/EBPα-ER followed by β-estradiol treatment. FACS analysis for CD11b 48 h after transfection. Numbers in dot blots indicate percentage of CD11b^+^ cells. The bars in the diagram summarize the mean ± SD of three independent experiments. *P* values are relative to control. **P ≤ 0.01, unpaired two-tailed *t*-tests. **e** FACS analysis of AnnexinV-stained K562-C/EBPα-ER cells overexpressing ERK5 or control. The bars represent the mean ± SD of three independent experiments. *P* values are relative to control. *P < 0.05, unpaired two-tailed *t*-tests

miR-143 expression and ERK5 protein levels were investigated during normal granulopoiesis of G-CSF-treated human CD34^+^ HSPCs and chemokine-induced granulopoiesis of K562-C/EBPα-ER, NB4, and U937 cells. Western blot analysis of ERK5 protein in G-CSF-treated CD34^+^ HSPCs showed a decline in ERK5 protein amount over a time period of 17 days followed by an increase at later time points, which inversely correlated with the expression of miR-143 at the investigated time points (Figs. 1b, 7a). In ATRA-treated NB4 and U937 cells as well as β-estradiol-treated K562-C/EBPα-ER cells we also observed an inverse correlation of ERK5 protein and miR-143 expression (Fig. 1c; Fig. 7b, Supplementary Fig. 7a). Furthermore, analysis of AML patient samples revealed an inverse correlation of miR-143 and ERK5 protein in malignant cells (Fig. 7c; Supplementary Table 6). Finally, transient overexpression of ERK5 in K562-C/EBPα-ER cells resulted in a decreased number of CD11b-positive cells and an increased number of apoptotic cells after β-estradiol-induced granulopoiesis (Fig. 7d, e; Supplementary Fig. 7b).

## Discussion

miRNAs, a class of regulatory molecules, have been frequently found to be dysregulated in human cancers^40^. Mature miRNAs act as negative gene regulators and have been shown to function both as tumor suppressors and oncogenes^41^. Recent studies showed that miR-143 is involved in a wide range of differentiation processes^21,42,43^ and carries out tumor suppressive functions in several cancer types^44–48^. However, at the moment only little is known about the role of miR-143 in myeloid differentiation and AML.

In this study, we found a strong induction of miR-143 expression during granulocytic differentiation in cell lines, primary CD34^+^ HSPCs as well as APL patients treated with ATRA, which could be supported by publications from Donahue et al. and Batliner et al^43,49^. Furthermore, we could show that miR-143 expression reached the highest levels in mature granulocytes and knockdown of miR-143 reduced the number of mature granulocytes in vivo. Our overexpression and knockdown experiments clearly demonstrate the impact of miR-143 during granulopoiesis. Our observations indicate a functional relevance of miR-143 in later stages of granulocytic differentiation. This is in contrast to previous findings, which describe miR-143 in early differentiation processes^50,51^. In solid tumors miR-143 is well described, but only a few studies regard the role of miR-143 in hematological malignancies^52–54^. In this study, we could show that overexpression of miR-143 leads to reduced proliferation and increased apoptosis of AML cells. Our observations are supported by data from Shen et al., which show that overexpression of miR-143 induces apoptosis in CML cells^55^. These data substantiate the tumor-suppressive function of miR-143 in the background of leukemia^53,56^. Further, we found that miR-143 expression significantly correlates with the survival of AML patients and is associated with good prognostic factors. These data show high miR-143 expression as a favorable prognostic factor in AML and substantiate a general role for miR-143 in prognosis, which is supported by data in solid cancers and from Elhamamsy et al. in AML^57–59^. Cancer patient prognosis is strongly affected by the response to chemotherapy. Several studies have suggested miRNAs as novel players in the development of chemotherapy resistance^60–62^. In this context, low miR-143 levels could be shown to be associated with chemotherapy resistance in solid cancers^63,64^, whereas, miR-143 overexpressing cells show enhanced sensitivity to chemotherapy in these cells^65–67^. In our study, we could show that treatment of AML cells, carrying an FLT3-ITD mutation, with phase III drug AC220 leads to the induction of miR-143 expression whereas stable miR-143 knockdown results in a diminished response to the treatment. Further, we could demonstrate that AML patients expressing high mir-143 levels show better response to 5-azacytidine-based chemotherapy. These data strongly support a role of miR-143 in the chemotherapy response of AML cells.

In our study, we identified miR-143 mRNA target pathways in myeloid cells by AGO-RIP-Seq. By in silico analysis, we could match several identified genes to basic cellular pathways. Beside pathways involving catalytic activity or nucleic acid and protein binding, members of the MAPK and mTOR pathway were notably highly enriched. Interestingly, the MEK/MAPK pathway is constitutively activated in the majority of AML subtypes and confers to uniformly poor prognosis^68,69^. In particular, ERK5, a member of the MAPK-family, was found to be enriched in the AGO-RIP-Seq. Besides its role in cell survival and proliferation, ERK5 is essential for growth of leukemic cells in vivo and one of the key players in chemotherapy resistance, including AML^34,37,38,70–72^. In the present study, we could identify ERK5 as direct target of miR-143 in hematopoietic cells, which is supported by previous publications showing ERK5 as direct target of miR-143 in solid cancers^30,66,73^. Moreover, the inverse correlation of miR-143 expression and ERK5 protein during granulopoiesis and in AML patient samples substantiate a functional interplay of miR-143 and ERK5 during normal granulocytic differentiation and in AML. Additionally, we could confirm functional involvement of ERK5 in myeloid differentiation^32,74^. These findings are in line with data from Wang et al. suggesting that ERK5 activity directs hematopoietic lineage selection towards the monocytic phenotype as well as with data from Carvajal-Vergara et al. showing that inhibition of ERK5 sensitizes cells to apoptosis^74,75^.

**Fig. 8:**
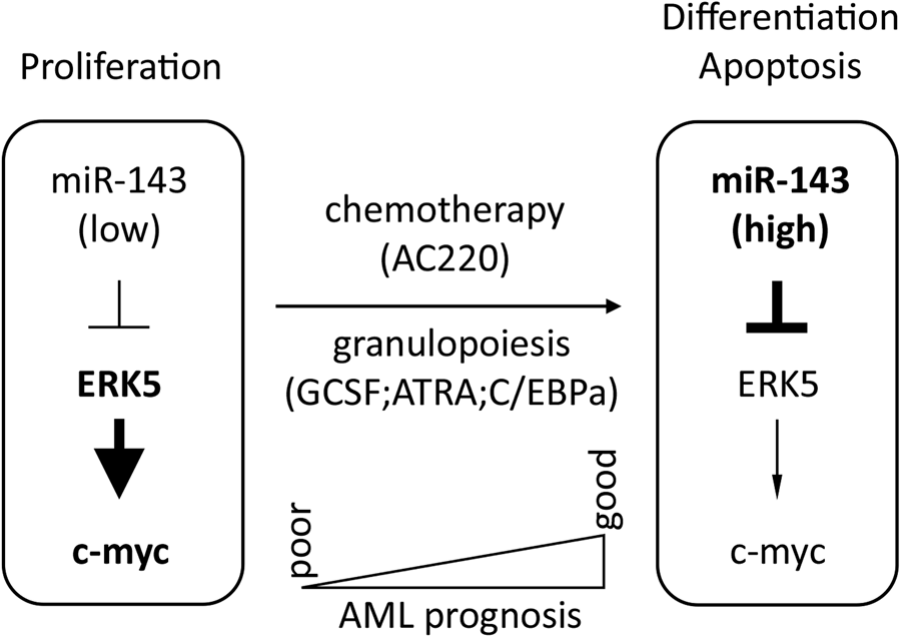
Schematic representation of a model for the role of miR-143 in granulopoiesis and in AML. During granulopoiesis, miR-143 expression is upregulated which in turn leads to repression of ERK5 and its downstream target c-myc resulting in myeloid differentiation. In AML, miR-143 is differentially expressed by various mechanisms. High miR-143 expression leads to better overall survival of AML patients whereas low miR-143 AML patients have a poor outcome. Overexpression of miR-143 in AML cells results in enhanced apoptosis and alters the susceptibility of those cells to chemotherapy

In summary, we show that miR-143 is an important regulator in myeloid differentiation by targeting ERK5. Along with these findings, we show that AML patients with high miR-143 expression show a better OS and benefit from AML treatment strategies (Fig. 8). It seems reasonable to suggest inhibition of the MAPK/MEK signaling by a miR-143 restoration approach as a novel therapeutic approach in AML therapy particularly with regard to chemotherapy resistance in AML^76–78^. Finally, miR-143 expression may function as a marker for creating personalized treatment strategies for patients suffering from this disease.

## Materials and methods

### Primary human samples

Patient samples were collected with written informed consent in accordance to the Declaration of Helsinki and under Ethics committee approval of each participating institution. AML patient samples were obtained as RNA or frozen bone marrow samples from the OSHO (East German Study Group Hematology/Oncology) patient sample collection at Leipzig University Hospital (Leipzig, Germany) and from Halle University Hospital (Halle, Germany). Blood cell samples from patients without hematopoietic diseases were obtained from the Halle University Hospital. All samples were karyotyped and molecular genetic analysis was performed. Hematopoietic CD34^+^ HSPCs cells were isolated from umbilical cord blood using CD34^+^ MicroBead kit (Miltenyi Biotec), as previously described^10^.

### Cells, cell culture, treatment, and transfections

NB4, U937, and 32D-FLT3-ITD cells were grown in RPMI-1640 medium (Life Technologies, Carlsbad, CA, USA) supplemented with 10% FBS and 1% penicillin/streptomycin/glutamine (P/S/G) (Life Technologies). K562-C/EBPα-ER were grown in RPMI-1640 medium supplemented with 10% charcoal-treated FBS (PAN-Biotech, Aidenbach, Germany), 1% P/S/G, and 1 μg/mL puromycin (Merck KGaA, Darmstadt, Germany). 293TN cells were grown in DMEM (Life Technologies) supplemented with 10% FBS. Primary human CD34^+^ HSPC cells were grown for 3 days in IMDM (Life Technologies) supplemented with 10% FBS, 50 ng/µL IL-6, SCF and FLT3-L. From day 4 on CD34^+^ HSPCs were grown in IMDM supplemented with 10% FBS, 10 ng/mL IL-3, 25 ng/mL SCF and FLT3-L. Granulocytic differentiation was induced with 100 ng/µL G-CSF starting from day 0 and every second day thereafter. All cytokines were purchased from Immunotools (ImmunoTools GmbH, Friesoythe, Germany). For differentiation of NB4 and U937, cells were induced with 1 µM ATRA (Merck KGaA) and DMSO as control. For differentiation of locked nucleic acid (LNA) transfected cells, 0.1 µM ATRA was used. Differentiation of K562-C/EBPα-ER cells was induced with 1 µM β-estradiol (Merck KGaA) and EtOH as control. 32D-FLT3-ITD cells were treated with 1 nM AC220 (Quizartinib) to inhibit FLT3 signaling.

Transfection of pcDNA6.2 plasmids, pcDNA3.1 plasmids, and LNAs was performed by electroporation (Amaxa Technology, Lonza) using cell specific Nucleofactor-Kits, according to the manufacturer’s instructions. For Luciferase assay lipofectamine LTX (ThermoFisher Scientific) was used according to the manufacturer’s instructions. Transfection efficiency was measured using flow cytometry (FACS) for fluorescent markers and was higher by 50% in all cells.

### miRNA expression profiling

In total, 500 ng RNA was extracted using TRIzol (ThermoFisher Scientific) method and used for small RNA library preparation with the TruSeqTM Small RNA Sample Prep Kit v2 (Illumina), according to the manufacturer’s instructions. The barcoded libraries were size restricted between 140  and 165 bp purified, and quantified using the Library Quantification Kit–Illumina/Universal (KAPA Biosystems), according to the manufacturer’s instructions. A pool of up to 12 libraries was used for cluster generation per lane. Library DNA at a concentration of 10 pM was clustered using an Illumina cBot, according to the SR_Amp_Lin_Block_Hybv8.0 protocol of the manufacturer. Sequencing of 50 bp was performed with an Illumina HighScan-SQ sequencer using version 3 chemistry and the version 3 flow cell according to the instructions of the manufacturer. Results were aligned to miRBase v21 and normalized. Median expression of each miRNA was set as threshold for low or high expression. Each experimental group was then analyzed for its level of expression compared to the calculated threshold.

### miRNA and mRNA detection by quantitative real-time PCR

Total RNA from cells was extracted using TRIzol. Briefly, cells were spun down and resuspended in 1 mL TRIzol and extracted with trichloromethane. RNA was then precipitated with isopropanol, washed with EtOH, and air dried. After resuspension of total RNA in water, the amount of RNA was measured using NanoDrop lite Spectrophotometer. miRNA and mRNA reverse transcription was done using 100 ng of RNA and MultiScribe^TM^ Reverse Transcriptase (ThermoFisher, #4311235 according to the manufacturer’s instructions. Reverse transcription of mRNA was performed using a 50:50 mixture of oligo dT and random hexamer primers. miRNA reverse transcription was performed using gene-specific primers according to the manufacturer’s instructions (Applied Biosystems). cDNA samples were used for qPCR analysis in a 1:10 dilution with gene-specific primers. qPCR reactions were performed on a MyiQ2 Two-Color Real-Time PCR Detection System (BIORAD) or an Applied Biosystems 7500 Real-Time PCR System (ThermoFisher) using SYBR green for mRNA samples and TaqMan probes for miRNA samples. RNU6B and RNU44 were used for normalization of human miRNAs, snoRNA-135 was used for normalization of mouse miRNAs, and GAPDH was used for normalization of mRNAs. All miRNA primers were obtained from Life Technologies. Specific mRNA primers were obtained from Biomers. Primer sequences for ERK5 and GAPDH are provided in Supplementary Table 7. Data analysis was done using 2-^ΔΔCt^ method.

### DNA constructs and cloning

The pcDNA6.2 expression vector and the pcDNA6.2 control vector, used for transient overexpression of miR-143 or control, were purchased from ThermoFisher Scientific. The pre-miR-143 sequence was cloned into the pcDNA6.2 expression vector according to the manufactures instructions (Supplementary Table 7). The pcDNA3.1-ERK5 was a kind gift from A. Martin-Villalba (IRB, Montpellier). The pcDNA3.1 control was obtained from ThermoFisher Scientific.

### Virus production and cell infection

Lentiviral vector (pLV-miR-143) for overexpression of miR-143 or an unspecific control (pLV-control) sequence was purchased from Biosettia (Biosettia Inc., San Diego, CA, USA). Both of them contained RFP reporter and showed puromycin resistance. Lentiviral vector for knockdown of miR-143 (miRZip-143) and the corresponding unspecific control (miRZip-control) vector were purchased from System Biosciences (System Biosciences, LLC, Palo Alto, CA, USA). Both of them contained GFP reporter and showed puromycin resistance. Pseudoviral particles were produced according to the manufacturer’s instructions using psPAX2, pMD2G in 293TN cells. Target cells were infected for 24 h with PEG-concentrated (System Biosciences) viral particles followed by puromycin selection or FACS sorting. Purity of transduced cells was analyzed using flow cytometry for expression of fluorescent markers and was > 90%, 7 days after selection or FACS sorting. Stable transduced cells were maintained in media supplemented with 1 μg/mL puromycin.

### Isolation of bone marrow precursor populations and granulocytes

Distinct murine bone marrow populations were sorted from C57BL/6NCrl mice, as previously described^10^.

### LSK cell isolation, manipulation, and transplantation into mice

Purification and culture of lineage^−^Sca-1^+^ cKit^+^ (LSK) cells was done as previously described^9,79^. Briefly, LSK cells were sorted from BM isolated from hips, legs, and spine of 12–17-week-old male C57Bl/6NCrl (Ly5.2^+^) mice. Sorted LSK cells were plated into StemSpan SFEM medium (Stemcell Technologies, Vancouver, Canada) supplemented with 10 ng/mL mIL-3, 20 ng/mL hIL-6, 100 ng/mL mSCF, 50 ng/mL mTPO, 100 ng/mL mFlt-3 ligand (all from PeproTech, Rocky Hill, NJ, USA), and 8 μg/ml polybrene (Sigma). LSK cells were then transduced with pseudoviral particles carrying miRZip-143 or miRZip-control lentivector (both containing GFP reporter). Virus was added at MOI of 20 for 6 h. Next, cells were washed twice with PBS, cultured for another 18 h, and then transplanted into recipient mice by tail vein injection. Congenic C57BL/6NCrl mice (Ly5.1^+^) were employed as recipients and they were lethally irradiated (two doses of 7.5 Gy, separated by 4 h). 5 × 10^4^ transduced LSK cells (Ly5.2^+^) were mixed with 5 × 10^5^ whole bone marrow cells as a support (Ly5.1^+^). Forty-eight hour after transduction, a non-transplanted aliquot of transduced cells was analyzed by flow cytometry for GFP positivity to assess efficiency of transduction. Recipient mice were bled and killed 3 weeks after transplantation and bone marrow was isolated. All mice that showed no proper engraftment (Ly5.2^+^ < 0.5%), did not express GFP, or showed general abnormalities in the GFP^−^ fraction were removed from final consideration. All mice experiments were approved by the Animal Care and Use Committee of the Institute of Molecular Genetics and were in agreement with local legal requirements and ethical guidelines.

### May-Grünwald/Giemsa staining, morphological analysis, and BM differential counting

For cytospin analysis, 5 × 10^4^–1 × 10^5^ cells were spun down on glass slides and stained with May-Grünwald and Giemsa staining solution (Dr. K. Hollborn & Söhne, Leipzig, Germany) according to the manufacturer’s instructions. Morphological analysis and manual leukocyte differential counts of bone marrow cells were performed using May-Grünwald/Giemsa stained cytospins. Cytospins were prepared from sorted bone marrow Ly5.2^+^ GFP^+^ cells from mice transplanted with LSK cells transduced with control or miR143 knockdown constructs. A minimum of 100 cells per cytospin was analyzed.

### Flow cytometry and fluorescence-activated cell sorting

For flow cytometry analysis, cells were prepared according to the manufacturer’s instructions and stained with specific antibodies. Labeled cells were analyzed on a BD LSR II or BD ARIA cytometer using CellQuest software (BD Biosciences). Final evaluation of data was done using FCS Express v6 (De Novo Software, Glendale, CA or FlowJo, FlowJo LLC).

Sorting of GFP^+^ cells and murine bone marrow subpopulations was performed as previously described^8^. For measurement of apotosis Annexin V Apoptosis Detection Kit I was used according to manufacturer’s instructions (BD Pharmingen #556547, #559763). All antibodies used for flow cytometry are listed in Supplementary Table 8.

### Cell growth assay

32D-FLT3-ITD cells stably transduced with pLV-miR-143 or control vector were plated in a density of 1 × 10^4^ cells/mL. Proliferation rate was ascertained by cell counting in a Neubauer chamber using Trypan blue staining for excluding dead cells.

### Argonaute2-RNA-immunoprecipitation (AGO-RIP) and next-generation sequencing (NGS)

RNA-immunoprecipitation was performed on K562-C/EBPα-ER cells stably overexpressing miR-143 or control using Imprint^®^ RNA Immunoprecipitation Kit (Sigma Aldrich), according to the manufacturer’s instruction. For each individual sample 3 × 10^6^ cells were used. 500 ng total RNA from AGO-RIP sample preparation was extracted using TRIzol. RNA library preparation was done with the TruSeq RNA Library Preparation Kit v2 (Illumina) according to the manufacturer’s instructions. Results were processed using TopHat and Cufflinks according to John L Rinn & Lior Pachter et al.^80^

### Luciferase assay

Luciferase assay constructs carrying the wild-type ERK5-3´ UTR and the ERK5-3´ UTR with the mutated miR-143 binding site between the luciferase ORF and the poly A tail were a kind gift from Prof. L. Fajas (Center for Integrative Genomics, CIG, Lausanne, Switzerland)^30^. The corresponding pGL3-control vector was purchased from Promega (Promega GmbH, Mannheim, Germany). Luciferase assays were performed in K562-C/EBPα-ER cells by co-transfection of the respective pGL3 construct, pRL (*Renilla* construct, and 200nmol miR-143 mimic molecules or negative control mimics (Dharmacon, Lafayette, CO, USA) using Lipofectamine LTX reagent. Luciferase activity was measured using the Dual Luciferase Reporter Assay (Promega) 24h after transfection according to the manufacturer’s instructions. Cotransfected pRL luminescence was used for normalization of luciferase activity.

### Western blot

Western blots were performed as previously described (9). Briefly, cells were harvested in standard RIPA buffer supplemented with protease and phosphatase inhibitor cocktails (Roche). The amount of protein was measured using Protein Assay Dye Reagent Concentrate (BIORAD) according to the manufacturer’s instructions using a Nanophotometer (Implen). Equal amounts of protein was loaded on a PAGE. Proteins were blotted semi dry with a Trans-Blot Turbo (BIORAD) according to the manufacturer’s instructions. Membranes were blocked with 5% BSA for 1 h at room temperature. If possible, the western blot membrane was cut to simultaneously probe for low- and high-molecular weight protein with 1st antibody at 4 °C overnight. Membranes were then washed probed with 2nd antibody and washed again. Probing of membranes with two different antibodies of the same size was done by stripping of the membrane and re-probing with antibody following the aforementioned procedure. Immunodetection was performed using WesternSure Blot Ultra- or WesternSure Blot Premium Substrate (Licor, Lincoln, Nebraska). Membranes were analyzed using a C-Digit Chemiluminescent Western Blot Scanner (Licor). Evaluation of data was performed using ImageJ software (NIH, Bethesda, MD, USA). All antibodies used for western blot are listed in Supplementary Table 8.

### Statistical analyses

All experiments were performed at least three times except for primary AML samples where at least two independent reverse transcription reactions and corresponding qPCRs were performed. Data are presented as means ± standard deviation. Statistical analyses were performed using SPSS (IBM, New York, USA) or Excel (Microsoft Corporation, Redmond, WA, USA). Student *t*-, Mann–Whitney *U*-, or log rank tests were performed to determine statistical significance of experimental results. A *p*-value of *P* ≤ 0.05 was considered significant (*) and a *p*-value of ≤ 0.01 (**) was considered as highly significant.

### Accession numbers

Data from next-generation sequencing have been deposited in the Gene Expression Omnibus (GEO) with the accession numbers GSE93549 and GSE93550.

## Electronic supplementary material

Supplemental Figures and Tables
