# Supplementary material for: MicroRNA-143 targets ERK5 in granulopoiesis and predicts outcome of patients with acute myeloid leukemia
Source: Cell Death Dis. 2018 Jul 26;9(8):814. doi: 10.1038/s41419-018-0837-x (PMC6062564; doi:10.1038/s41419-018-0837-x)
Supplement: Supplementary file 1 — Supplemental Figures and Tables [file 41419_2018_837_MOESM1_ESM.pdf]

Supplement to Figure 1.

a

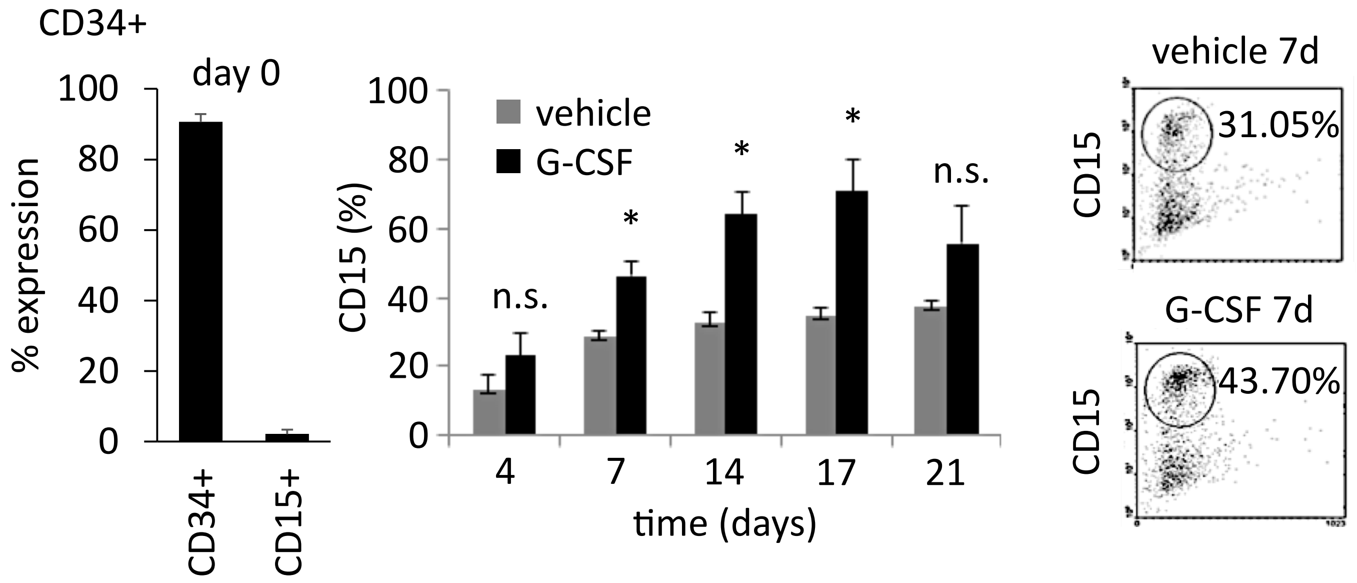

b

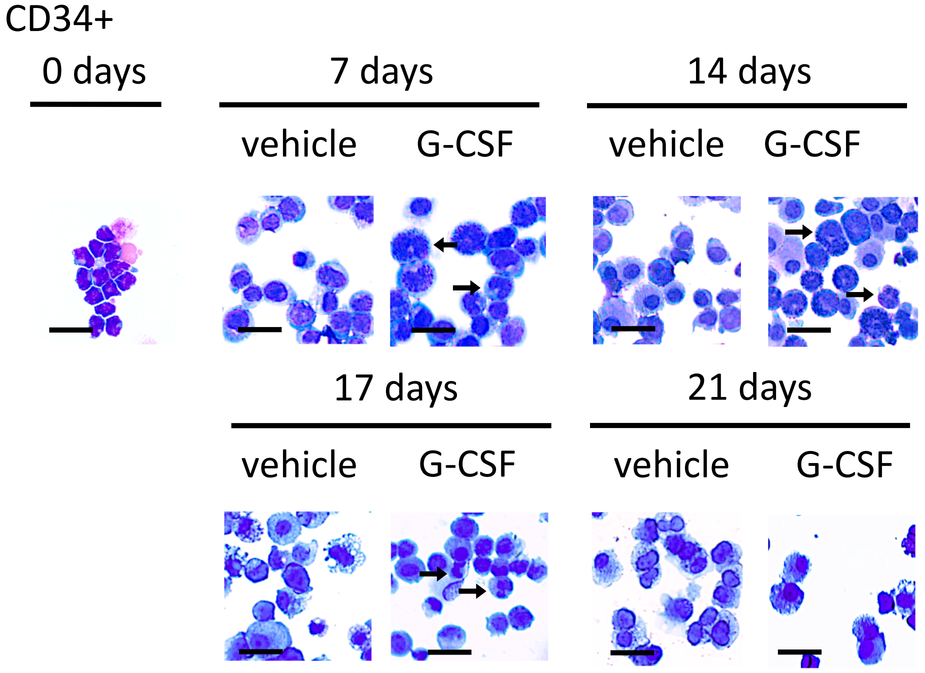

c

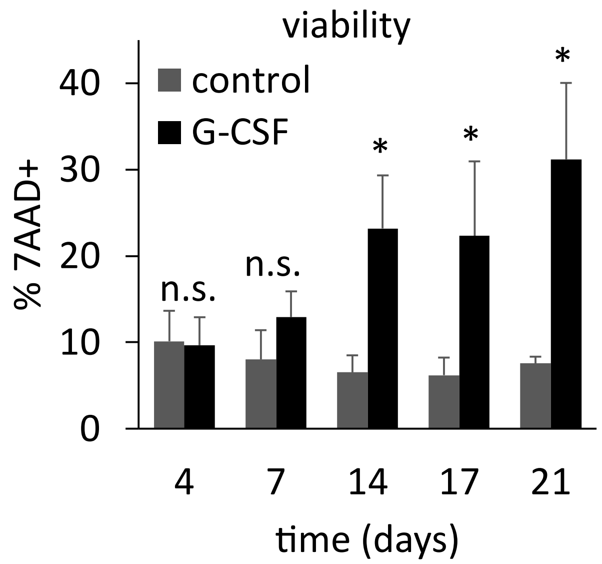

Supplemental Figure 1. (related to Figure 1) MiR-143 is upregulated during granulocytic differentiation of G-CSF treated Cd34+ HSPCs. a) FACS analysis of CD34 and CD15 expression of freshly isolated CD34+ HSPCs (left diagram). FACS analysis of CD15 expression in G-CSF or vehicle (H_2O) treated human CD34+ HSPCs as representative FACS blots at day 7 (right) and as summary of 3 independent experiments over time (middle). **b)** Giemsa/May-Grünwald-stained cytospins at indicated time points. Arrows indicate nuclei with granulocyte morphology. Scale bar represent 20**µ**m **c)** QPCR analysis for miR-143 expression in these cells at the same time points, represented as summary of 3 independent experiments +/-SD. *P<0.05; unpaired two-tailed *t* tests.

Supplement to Figure 1.

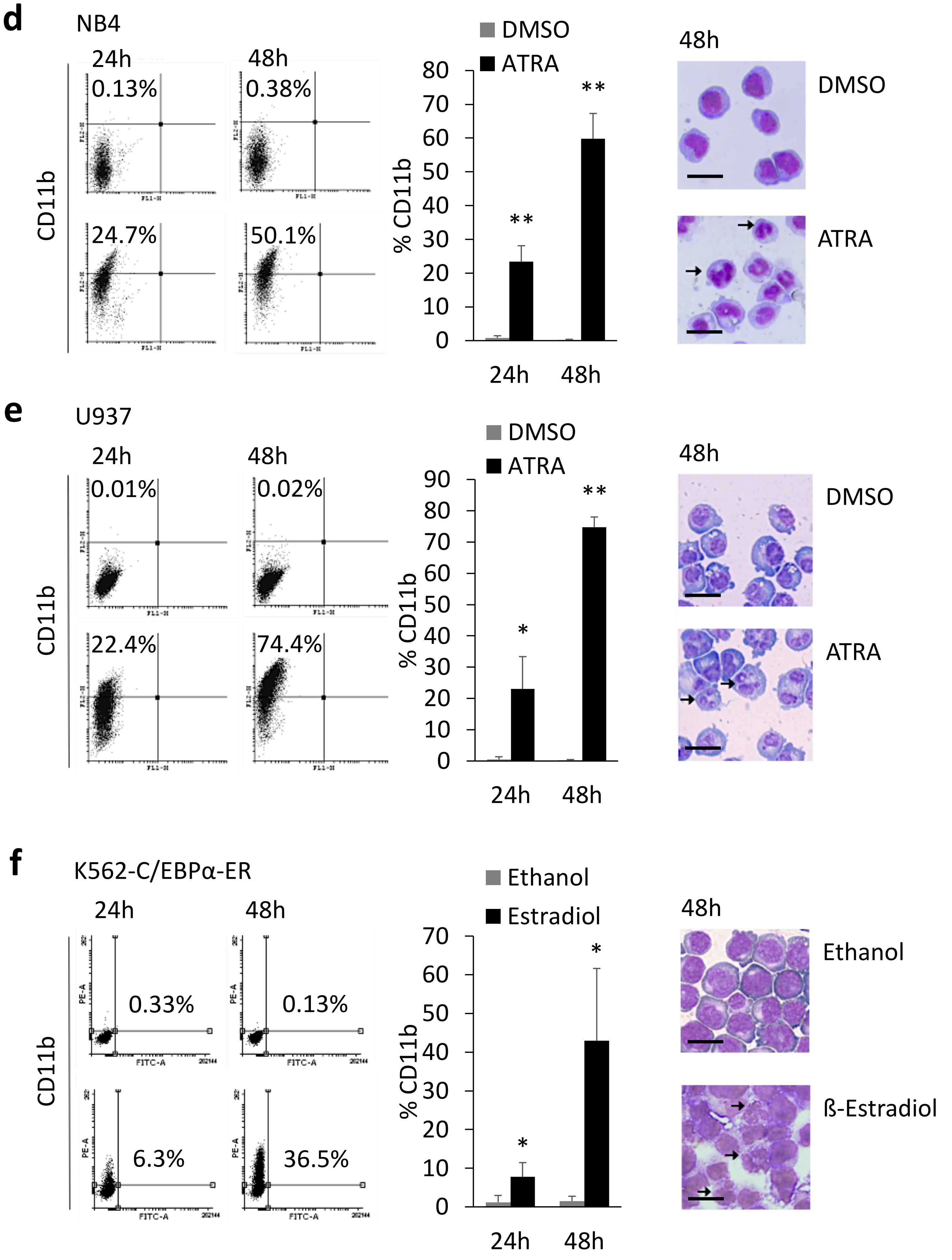

Supplemental Figure 1. (related to Figure 1) FACS and qPCR analysis of myeloid cells undergoing granulocytic differentiation. Representative FACS analysis of CD11b expression of NB4 cells (**d**), U937 cells (**e**) and K562-C/EBPa-ER cells (**f**) treated with 10^{-6}M ATRA (NB4; U937) or 10^{-6}M ß-Estradiol (K562-C/EBPα-ER) and DMSO or Ethanol as control. Analysis was performed at indicated time points. Diagrams next to the dot blots represent the mean of 3 independent experiments +/-SD. **P<0.01; *P<0.05; unpaired two-tailed *t* tests. Giemsa/May-Grünwald-stained cytospins of NB4, U937 and K562-C/EBPα-ER. Arrows indicate nuclei with granulocyte morphology. Scale bar represent 20μm.

Supplement to Figure 2.

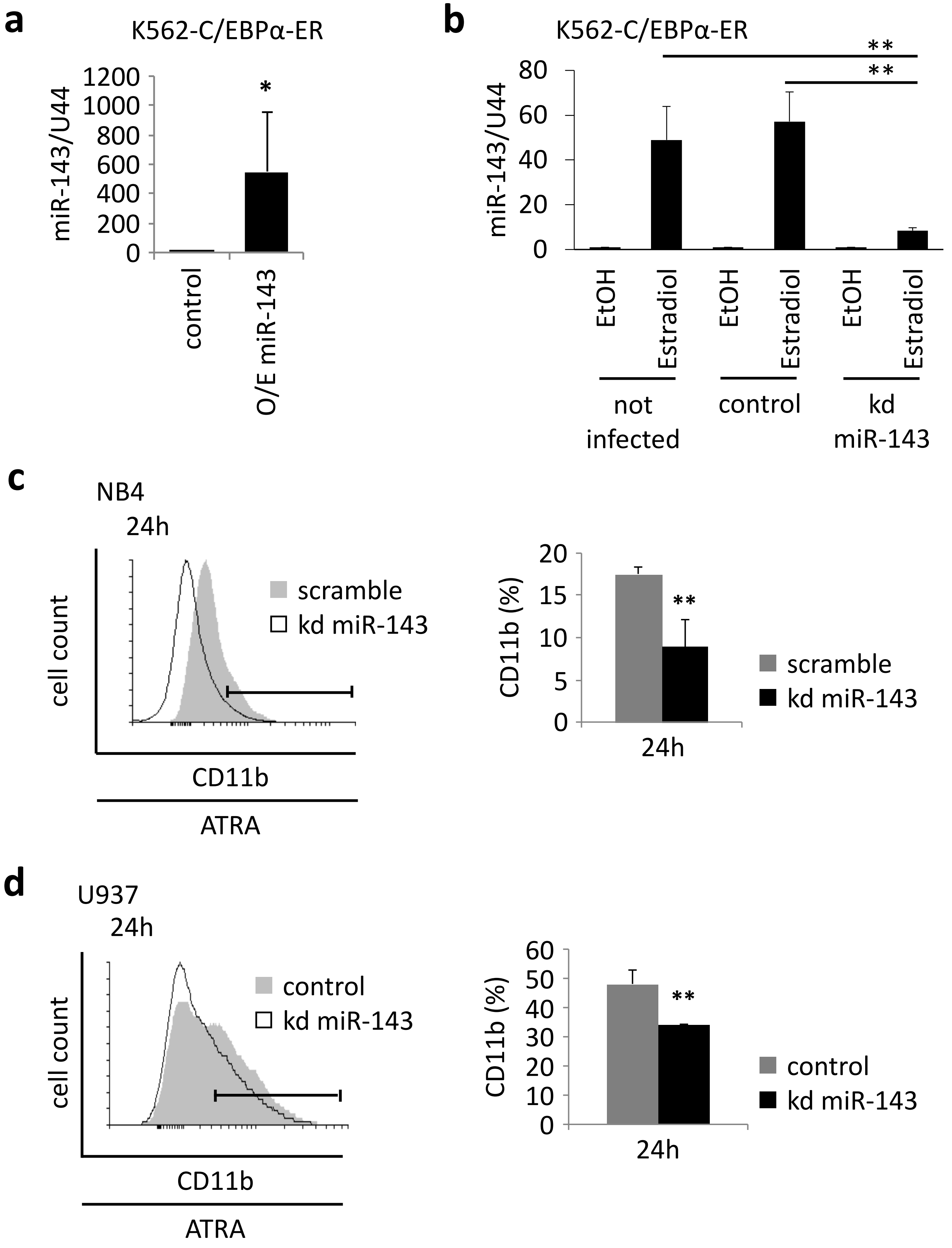

Supplemental Figure 2. (related to Figure 2) Overexpression and knock-down of miR-143 in myeloid cell lines. a) Transient overexpression of miR-143 or control (pcDNA6.2). QPCR analysis for miR-143 was performed 24h after transfection. **b)** Stable knock down of miR-143or control (miR-ZIP). QPCR analysis for miR-143 was performed in pyromycin selected cells and not infected cells treated with ß-estradiol for 72h. Normalization was done by U44. **c)** FACS analysis of CD11b expression in NB4 cells transfected with Locked Nucleic Acids (LNAs) specific for miR-143 or unspecific scramble LNAs 24h after transfection and treatment with ATRA. **d**) FACS analysis of CD11b expression in U937 cells stably expressing miR-ZIP-143 or miR-ZIP-control oligos 24h after ATRA treatment. Diagrams next to the histograms represent the mean +/-SD from 3 independent experiments. *P<0.05; **P<0.01, unpaired two-tailed *t* tests.

Supplement to Figure 3.

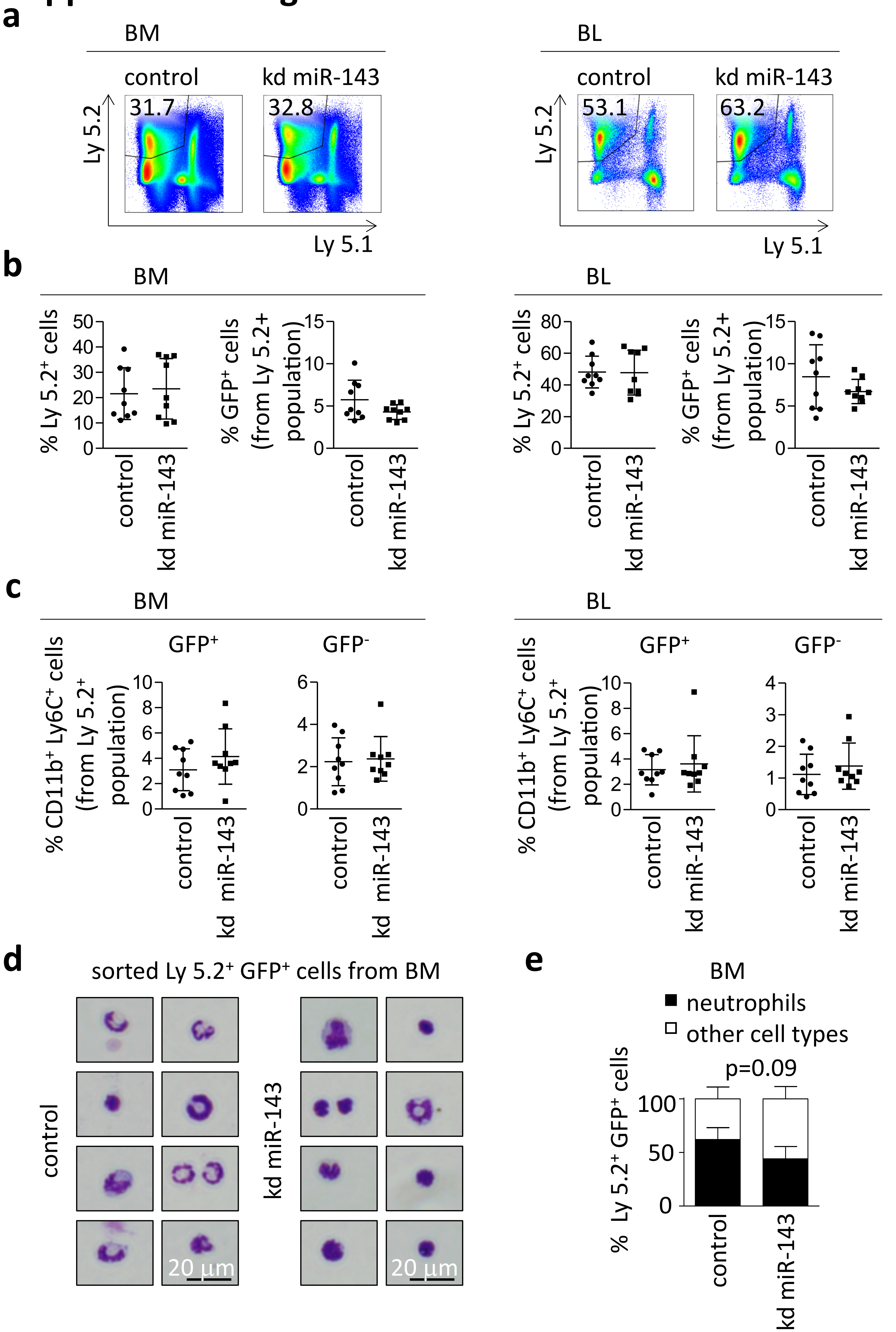

Supplemental Figure 3 (related to Figure 3). Transplantation of LSKs with stable knock down of miR-143 show comparable total engraftment of Ly 5.2^{+} cells and contribution of GFP^{+} donor cells to the hematopoietic system of the recipients. **a)** Representative plots of flow cytometry analysis of total engraftment from BM and BL of recipient mice transplanted with LSK cells transduced with control (ctrl, left panels) or miR143 knockdown (kd miR-143, right panels) constructs. Cells were stained for Ly 5.2 (donor derived, y axis) and Ly 5.1(recipient derived, x axis) surface markers. Black boxes indicate percentage of Ly 5.2^{+} Ly 5.1^{-} cells. **b)** Total engraftment of Ly 5.2^{+} cells (left panel) and contribution of GFP^{+} donor cells (right panel) in the BM (left part) and BL (right part) of recipient mice mice transplanted with LSK cells transduced with control (ctrl) or miR143 knockdown (kd miR-143) constructs. Y axis indicates the percentage of Ly5.2^{+} cells (left panel) or percentage of GFP^{+} cells within Ly5.2^{+} gate (right panel). **c)** Quantification of donor derived monocytes in the BM (left part) and BL (right part) of recipient mice in GFP^{+} (upper graphs) and GFP^{-} (lower graphs) fraction. Y axis indicates the percentage of CD11b^{+}/Ly6C^{+} monocytes. Bars represent the mean +/-SD from two independent experiments, n=9 (control) or 10 (kd miR-143) mice, differences were not significant **d)** Representative pictures of the cell morphology analysis from Ly 5.2^{+} GFP^{+} sorted bone marrow cells from mice transplanted with LSK cells transduced with control (control, left panel) or miR143 knockdown (kd miR-143, right panel) constructs. Cells were cytospun and stained with May/Grünwald-Giemsa. Mature neutrophils have characteristic segmented nuclei. Scale bar 20 µm. **e)** Quantification of differential cell counting analysis of cytospins presented in panel d. At least 100 leukocytes were counted for each cytospin. Y-axis indicates the percentage of neutrophils. Bars represent the mean +/-SD from two independent experiments, n=3 (control) or 4 (kd miR-143) mice, P=0.09, unpaired two-tailed *t* tests were used to assess statistical significance.

Supplement to Figure 4.

a

characteristics	low miR-143	high miR-143	P
age, years<br>median<br>range	 61<br>19-72	 61<br>29-73	0.39
gender, no<br>male<br>female	 16<br>20	 20<br>21	0.82
**WBC, x10^9/l**<br>**median**<br>**range**	 22.4<br>1-385	 3.65<br>0.7-118	**0.008**
**PB blasts, %**<br>**median**<br>**range**	 48<br>2-97	 21<br>0-97	**0.02**
BM blast, %<br>median<br>range	 75<br>26-95	 62.25<br>10.95	0.5

b

non-myeloablative (NMA) conditioned
transplanted AML patients

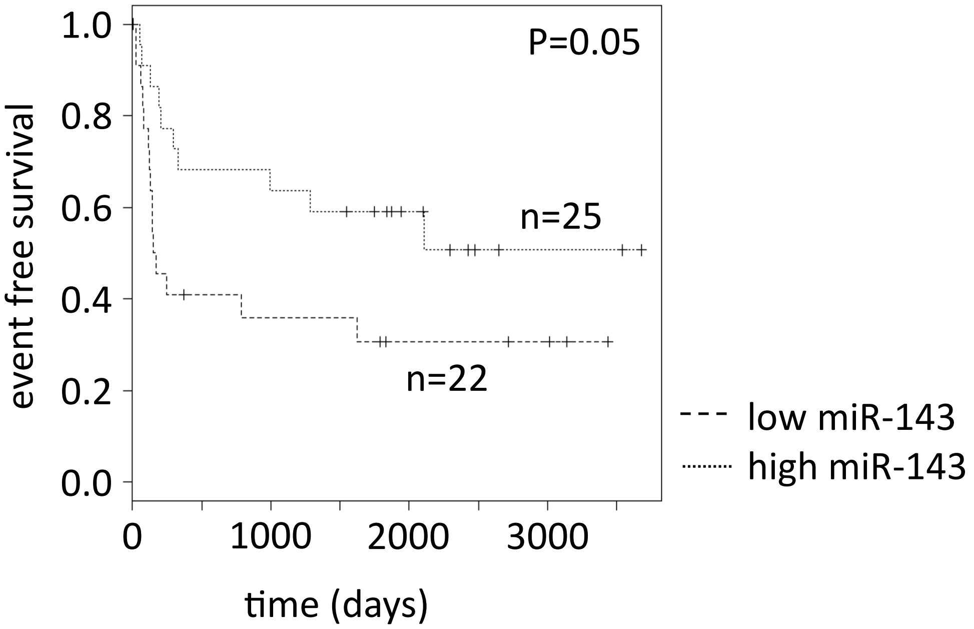

Supplemental Figure 4. (related to Figure 4) a) Patient sample characteristics of AML patient sample cohort from the OSHO data base. Characteristics were analyzed according to the miR-143 expression. Statistic significant parameters are signed bold. **b)** Kaplan-Mayer curve for the survival of the OSH patient sample cohort according to the expression of miR-143. MiR-143 expression was divided by the median cut. Mann-Whitney-U: P=0.05.

Supplement to Figure 5.

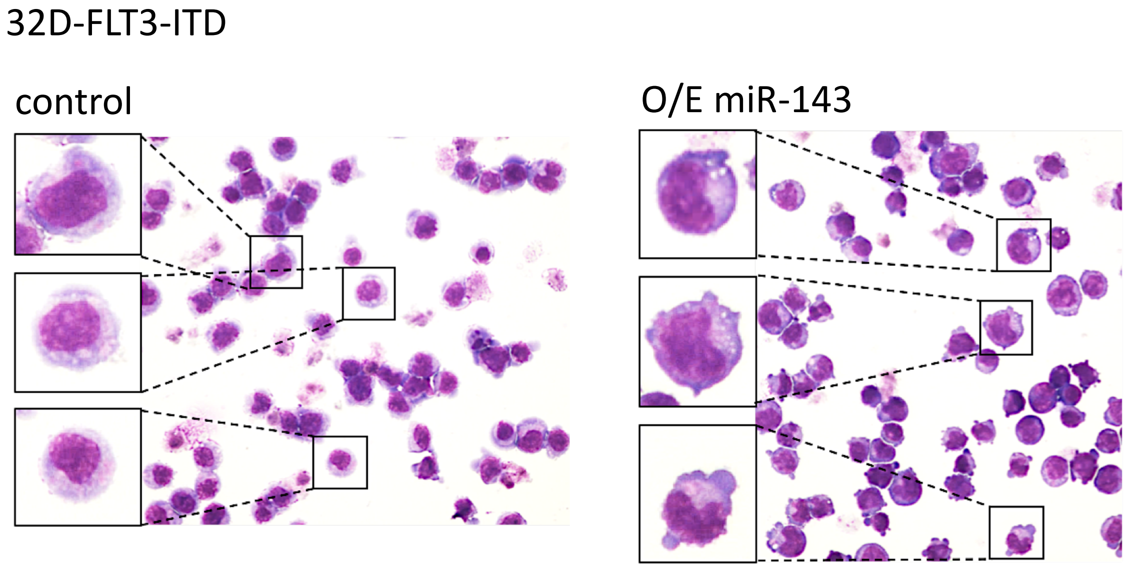

Supplemental Figure 5. (related to Figure 5) Morphological analysis of 32d-FLT3-ITD cells stably overexpressing miR-143 or control. Marked cells are the cells represented in Figure 5c in the manuscript. Scale bar represent 10μm.

Supplement to Figure 6.

a

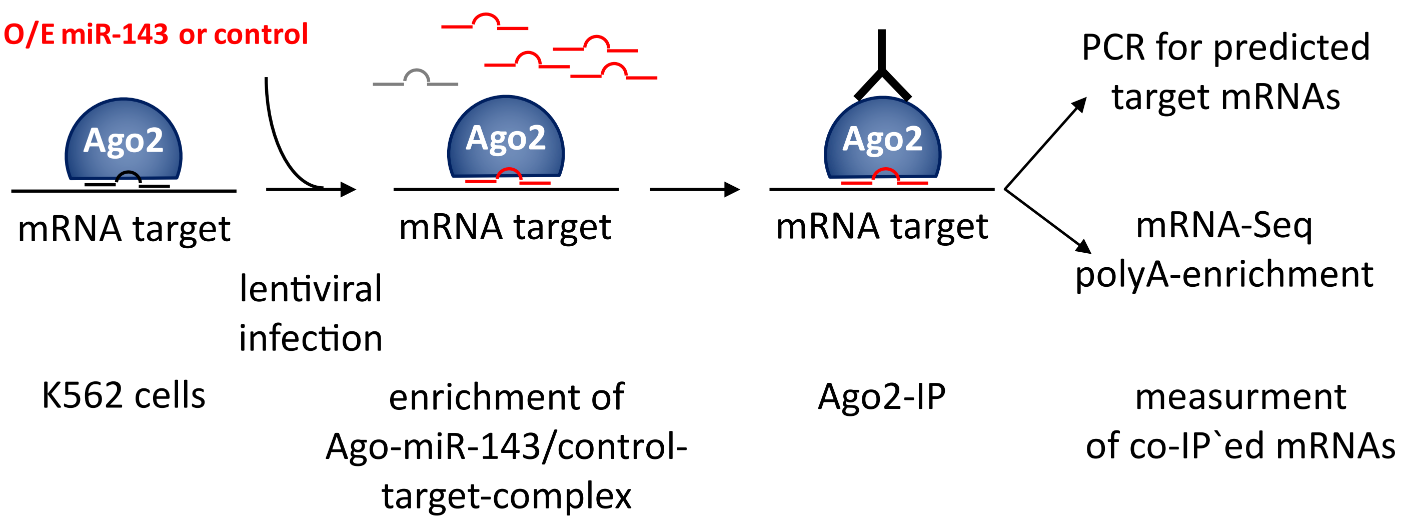

b

```
MAPK7 3' UTR (ERK5; position 120-126)   5'  ...GAUUAUUCUGCAGGU--UCAUCUCA...3'
                                                          |||     |||||||
hsa-miR-143                             3'           CUCGAUGUCACGAAGUAGAGU     5'
```

c

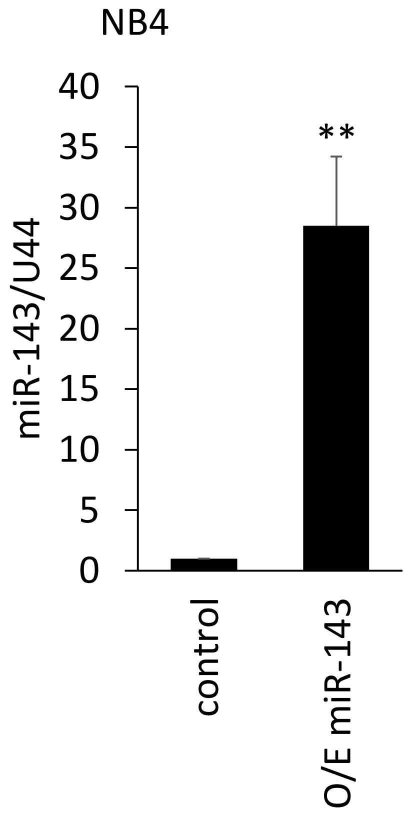

d

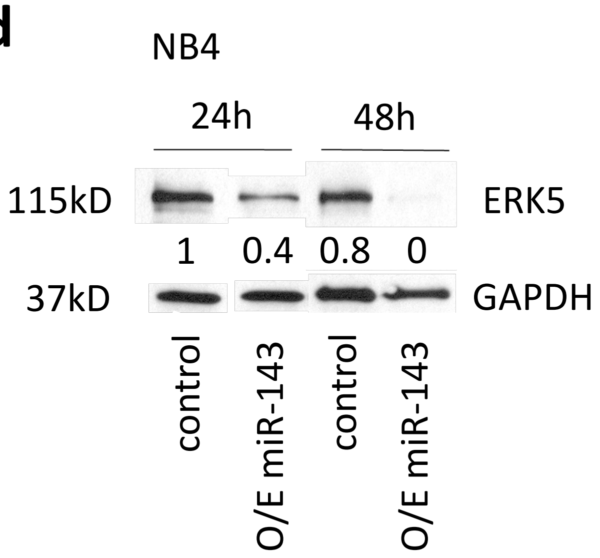

Supplemental Figure 6. Identification of miR-143 target mRNAs. (related to Figure 6) a) Schematic representation of the AGO-RIP-procedure. **b**) Schematic representation of the conserved miR-143 binding site in the 3´UTR of ERK5 mRNA. Numbers in front of the sequence represent the nucleotide position relative to the termination codon of the human ERK5. **c**) QPCR analysis of miR-143 expression in NB4 cells after transient overexpression of miR-143 or control (pcDNA6.2). Bars represent the mean of 3 independent experiments +/-SD. **P<0.01; unpaired two-tailed *t* tests. **d**) Western blot analysis of ERK5 and GAPDH (control) in NB4 cells overexpressing miR-143. The western blot pictures represent one example of 3 independent experiments. Numbers below the blots represent the ratio of ERK5 to the respective GAPDH control.

Supplement to Figure 7.

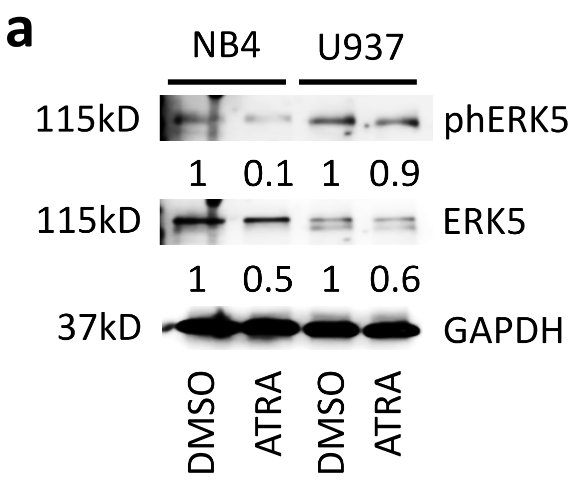

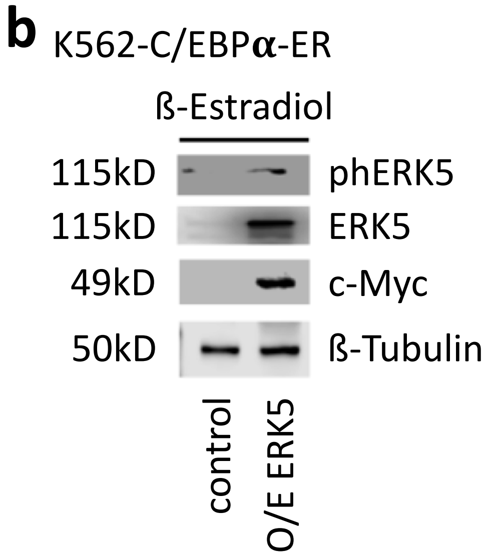

Supplemental Figure 7. ERK5 protein and downstream signaling is modified upon granulocytic differentiation, ectopic overexpression and inhibition by XMD8-92. (related to Figure 7) Western blot analysis of phosphorylated ERK5, ERK5, c-Myc and GAPDH **a)** in ATRA treated NB4 and U937 cells (48h). **b)** in K562-C/EBPa-ER cells overexpressing ERK5 or control (pcDNA3.1) and treated with ß-Estradiol (48h). The western blot pictures represent one example of 3 independent experiments. Numbers below the blots represent the ratios to the respective control (GAPDH or ß-Tubulin).

Supplemental Table 1. APL patient characteristics. (related to Figure 1d) APL patient characteristics used for miR-143 expression analysis.

APL patient number	diagnosis	Morphology by FAB	age (years)	date of sample-taking	status	material source
#1	t(15;17)	M3	54	02.04.2009	diagnosis	BM
				18.09.2009	remission	BM
#2	t(15;17)	M3	67	23.02.2007	-	PB
				18.02.2010	-	PB
#3	t(15;17)	M3	53	16.06.2009	diagnosis	BM
				25.09.2009	repetition	BM
#4	t(15;17)	M3	57	25.05.2010	diagnosis	BM
				07.06.2010	-	BM

FAB: French-American-British; t: translocation; BM: bone marrow; PB: peripheral blood.

Supplemental Table 2. TCGA AML patient characteristics. (related to figure 4 a;b;c) Patient characteristics used to analyze overall survival, MRC risk group classification, PB/BM blast count and WBC according to miR-143 high or low expression.

characteristics	low miR-143	high miR-143	P
age, years			
median	60	57	0.34
range	18-88	23-82	
Gender, no			
male	51	50	
female	43	44	
**WBC, x10^9/l**			
median	40	10	**< 0.001**
range	1-298	1-114	
**PB blasts, %**			
median	49	24.5	**< 0.001**
range	0-98	0-91	
**BM blasts, %**			
median	78	60	**< 0.001**
range	30-99	11-100	
MRC, no			
Favorable	5	30	
Intermediate	66	43	
Adverse	21	21	

WBC: white blood cell count; PB: peripheral blood;
BM: bone marrow; MRC: Medical Research Council

Supplemental Table 3. Characteristics of AML patients with/ without FLT3-mutations. (related to Figure 4d) AML patient characteristics used for miR-143 expression analysis.

AML patient number	karyotype	FLT3 status	age (years)	gender	material source
#1	normal	ITD	66	female	bone marrow
#2	normal	ITD	67	male	bone marrow
#3	normal	ITD	61	male	bone marrow
#4	normal	ITD	59	male	bone marrow
#5	normal	ITD	48	female	bone marrow
#6	normal	WT	52	male	bone marrow
#7	normal	WT	72	male	bone marrow
#8	normal	WT	63	male	bone marrow
#9	normal	WT	66	female	bone marrow
#10	normal	WT	69	female	bone marrow
#11	normal	WT	65	female	bone marrow
#12	normal	WT	55	male	bone marrow
#13	normal	WT	65	male	bone marrow
#14	normal	WT	72	female	bone marrow
#15	normal	WT	66	female	bone marrow

Supplemental Table 4. Characteristics of AML patients used for stable overexpression of miR-143 (related to Figure 5g). AML patient characteristics used to overexpress miR-143 for Annexin V FACS analysis.

	FAB	age	gender	karyotype	blast count (%)	source
AML #16	M2	57	male	43,XY,?-3,inv(3)(q21q26),der(4)t(4;7)(q2?5;?) der(?5;17)(?;q10),-7, der(7;15)(q10;q10),der(8)t(3;8)(?;q?13),-11,del(12)(q23q24)[cp6]/45,X,-Y[7]/46,XY[7]	37	bone marrow
AML #17	M2	70	male	46,XY[30]	81	peripheral blood

Supplemental Table 5. 5-azacytidine treated AML patient characteristics. (related to Figure 5h) AML patient characteristics used to analyze treatment response to 5-azacytidine according to miR-143 high or low expression.

patient type	FAB	gender	age	treatment	blastcount t0	blastcount t15
non-responders	M2	m	79	aza	34	10
non-responders	unknown	m	62	aza	75	75
non-responders	M2	m	63	aza	24	90
non-responders	M4	f	68	aza	69	21
non-responders	M7	f	70	aza	70	67
non-responders	M1	f	70	aza	70	80
non-responders	M2	m	66	aza	80	60
non-responders	M1	m	73	aza	75	90
non-responders	M4	f	62	aza	90	35
non-responders	M4	m	68	aza	60	70
non-responders	M5	f	66	aza	83	unknown
non-responders	M4	m	72	aza	57	70
non-responders	M1	f	72	aza	80	50
non-responders	M4	m	73	aza	81	12
non-responders	M2	m	65	aza	64	11
responders	M2	m	77	aza	20	-
responders	M4	f	76	aza	30	-
responders	M2	m	74	aza	50	-
responders	M1	m	78	aza	25	-
responders	M4	m	70	aza	80	-
responders	M5	f	75	aza	70	-
responders	M5	m	61	aza	80	-
responders	M0+M2	m	68	aza	52	-
responders	M5	m	70	aza	50	-
responders	M1	f	64	aza	95	-
responders	unknown	m	76	aza	10	-

FAB: French-American-British; NK: f: female; m: male; aza: 5-azacytidine.; t: time in days

Supplemental Table 6. Characteristics of AML patients used for ERK5 Western blot and miR-143 expression. (related to Figure 7c) AML patient characteristics used to analyze ERK5 protein level.

AML patient number	diagnosis	morphology by FAB	age	gender	material source
#18	NK	M5	65	f	BM
#19	Flt3-mut.	M1	40	f	BM
#20	Flt3-mut.	M4	74	m	BM
#21	Flt3-mut.	M5a	40	f	BM
#22	Flt3-mut.	-	66	m	BM
#23	CK	M5	73	m	BM
#24	CK	M5	56	m	BM

FAB: French-American-British; NK: normal karyotype; CK: complex karyotype; f: female; m: male; BM: bone marrow

Supplemental Table 7. Primer list.

Primers for PCR amplification	
ERK5 forward	5`ACG AGT ACG AGA TCA TCG AGA CC 3`
ERK5 reverse	5`GGT CAC CAC ATC GAA AGC ATT AGG 3`
GAPDH forward	5`ACC ACA GTC CAT GCC ATC AC 3`
GAPDH reverse	5`TCC ACC ACC CTG TTG CTG TA 3`
Primers for cloning	
miR-143-sense	5´TGCTGGGTGCAGTGCTGCATCTCTGGTCAGTTGGGAGT CTGAGATGAAGCACTGTAGCTC3´
miR-143-antisense	5´CCTGGAGCTACAGTGCTTCATCTCAGACTCCC AACTGACCAGAGATGCAGCACTGCACCC3´

Supplemental Table 8. Antibody list.

**FACS antibodies**
Ly5.2 (Biolegend #109827, 109829)
Ly5.1 (Biolegend # 110721)
Ly6G (Biolegend # 127613)
Ly6C (Biolegend # 128017),
CD11b (Biolegend # 101207, BD Pharmingen# 555388)
CD15 (BD Pharmingen #555402, #555401)
CD34 (BD #345801, #345802)
anti-mouse Lineage cocktail (Biolegend #133310)
c-kit (Biolegend #105807)
Sca-1 (Biolegend #108111)
B220 (Biolegend #103211)
Hoechst 33258 (Sigma #861405)
**Western blot antibodies**
rabbit mAb Anti-ERK5 (#3372, Cell Signaling)
rabbit pAb Anti-pERK5 (#3371, Cell Signaling)
rabbit pAb Anti-c-myc (#9402S, NEB)
rabbit pAb Anti-GAPDH (sc-25778, Santa Cruz Biotech).
donkey-anti-rabbit-HRP (NA934V, GE-Healthcare)
goat-anti-rabbit (sc-2004, Santa Cruz Biotech.)
